# From fixing to connecting—developing mutual empathy guided through movement as a novel path for the discovery of better outcomes in autism

**DOI:** 10.3389/fnint.2024.1489345

**Published:** 2025-04-02

**Authors:** Anat Baniel, Eilat Almagor, Neil Sharp, Ohad Kolumbus, Martha R. Herbert

**Affiliations:** ^1^Anat Baniel Method, Inc., San Rafael, CA, United States; ^2^The Jerusalem Academy of Music and Dance, Jerusalem, Israel; ^3^ABM^®^ NeuroMovement^®^ Center of Marin, San Rafael, CA, United States; ^4^Higher Synthesis Foundation, Lobelville, TN, United States; ^5^Complementary Healthcare Advanced Research and Leadership Institute, Life University, Marietta, GA, United States

**Keywords:** movement, signal-to-noise ratio, autism, neuroplasticity, learning, connection, transformative change, empathy

## Abstract

This article presents the theoretical foundation of two well established movement-based methods that represent a fundamental departure from most current interventions and are applied globally with children and adults experiencing diverse motoric, cognitive, and social challenges as well as with high functioning individuals: the Feldenkrais method and Anat Baniel Method^®^ NeuroMovement^®^. These methods are based on leveraging neuroplasticity through the utilization of movement, not as “exercise” or externally imposed motor sequences, but as a means for effective, *two-way felt communication* with the recipient and their brain. Through *connecting with the recipient, starting where they are–motorically, emotionally, and cognitively*, we follow their *unique responses, moment-by-moment*, creating a dance-like dyadic process of self-discovery that mimics the spontaneous, organic way typically developing children play, learn, and grow. Practitioners in these methods, by joining and creating mutual connection with the recipient, help turn the subjective experience of the recipient into a reliable means of attaining spontaneous, mutually generated emergent learning in the recipient. In this process the autonomy of the recipient is respected and enhanced. Our work will be described through direct applications to autism seen as a neuro-motor-sensing disorder where those challenges can be transcended through the dyadic dance embodied in our techniques. Since 87% of children with autism spectrum disorder have significant movement challenges, we propose that movement, as a means for effective two-way communication with the child and their brain, needs to play a central role in autism intervention. In this article we outline how our interventions take place through case studies, vignettes and discussion, separately for each of the two methods. This article will also include recommendations for conducting investigations that characterize some of the basic components of these two methods, utilizing experimental designs and recently developed technologies and biometrics that generate unique individual profiles of both the receiver and the provider of the intervention, and of the interbrain synchrony, correlate them with changes in movement organization, cognitive functioning and coherence, and track changes in the signal-to-noise ratio. These methods should enable refinement and scalability of tracking and assessing the mechanisms and effectiveness of the interventions.

## 1 Introduction

Currently autism spectrum disorder (ASD) is defined as a *developmental disorder* that primarily impacts the *social, behavioral*, and *communication* domains (Lord et al., 2000; American Psychiatric Association, 2013). Movement challenges, when noted, are generally considered *comorbid*, i.e., not a core part of the disorder. In recent years, however, research by the Simons Foundation involving thousands of children on the spectrum has shown that 87% of children who meet criteria for ASD have significant movement challenges (Bhat, 2020, 2021), and the severity of the ASD symptoms has been shown to correlate with the severity of the motor challenges (Bhat, 2021). These findings call for a re-evaluation of the role of movement in autism as a contributor both to the condition itself, and to intervention that leads to transformative outcomes.

### 1.1 Movement

We propose that movement is an important underlying component of autism, because (1) movement disturbances have been identified in children far earlier in development than when challenges in social, behavioral, and communication domains have been identified (Bauman, 1992; Teitelbaum et al., 1998; Posar and Visconti, 2022; Torres et al., 2023); and (2) as we will discuss in detail, movement needs to play a central role in interventions with the children on the spectrum: not as “exercise” or externally imposed motor sequences, but as a means for effective two-way communication with the child and with their brain. As we will describe in detail, this communication is done through *connecting with the child starting where they are. We follow their unique responses continuously* and introduce a process that mimics the spontaneous and organic way typically developing (TD) children learn and grow.

In this article we will present the foundations, practices, scientific implications, and some clinical applications of two related but distinct *movement-brain-learning-based* practices: the *Feldenkrais method (FM)* (Feldenkrais, 1972a,b,c) and *Anat Baniel Method^®^ NeuroMovement^®^ (ABMNM^®^)* (Baniel, 2012a; see Supplementary Table 1-Table of Contents and Supplementary File 1-ABMNM “From Fixing to Connecting”. Both methodologies operate under assumptions and ways of intervention that are very different from current and more conventional approaches. The goal of most movement interventions in the medical and rehabilitation therapies (for example, *physical therapy*), is to improve or correct impairment of some part of the body, caused by injury or medical conditions. The intention therein is to reduce dysfunction and dependency by trying to “make” the individual perform in more “normal,” “productive,” or “neurotypical” ways, mostly focused on the area of injury or dysfunction.

The functioning human, including their body, is however a highly dynamic, complex, interconnected set of linkages organized and managed by the brain, with many degrees of freedom (Bernstein, 1967; Feigenberg and Linkova, 2014), whereby motions of one segment affect the other segments, as well as thinking and feeling, in interdependent ways that the practitioner and the recipient need to discover together using a holistic approach. ABMNM and FM provide a holistic approach that aims to account for the whole system and its complexity in a dynamic way. Both the FM and ABMNM have been applied for decades, for enhancement as well as repair, across the entire range of human capabilities, spanning from major challenges such as autism and stroke, to enhancing peak performance in sports, music, intellectual development, and the arts.

Here we share with our readers our insights on ways of creating connection, coordination and togetherness of two bodies and two brains, that of the practitioner and that of the recipient, that generate new information for the recipient to work with. They share a common space and operate as “one system” during their dyadic interaction. Motions in that shared space reveal a process of exploration and self-discovery of movement and sensations that heighten self-awareness and offer new avenues for the recipient to spontaneously learn new ways of moving, thinking, and interacting with others.

### 1.2 Learning

Learning is profoundly intertwined with movement. Learning *starts with movement in the womb*, along with its associated sensations which provide information for the brain to form itself and expand its capabilities. Human beings are fully dependent on learning for the development of their voluntary motor, cognitive, emotional, and inter-personal functioning. When newborns enter and interact with the external world, they experience the pull of gravity separate from their mother for the first time. Their initial movements are utterly unskilled, mostly reflexive, and random. These movements, however, have continuous consequences related to the constant pull of the gravitational force, which the child experiences through sensations and outcomes. During a very lengthy process, unparalleled in the animal kingdom, the child organizes and integrates their sensory and proprioceptive experiences, and thereby learns to control their actions and to generate intentionality and awareness, a process Feldenkrais named “organic learning” (Feldenkrais, 1981b). This process typically leads to mastering uniquely human capabilities of upright balancing and walking on two legs, talking, abstract thinking and much more. In our experience, children on the autism spectrum often have significant challenges in forming a successful foundational relationship with the gravitational pull which we understand leads to later significant movement and other challenges common in ASD. This invites us to shift our attention, when working with the child with ASD, from trying to fix the behavioral and psychological symptoms (which may well be downstream of the movement challenges) to focusing on working with movement and its associated sensations, starting where the child is, as an avenue into the process of *progressive* differentiation and *progressive* integration–the way organic learning takes place with typically developing children. This organic learning process helps the child “learn how to learn” and it can then generalize to many other domains of functioning.

In the dyadic processes of FM and ABMNM, “organic learning” is an internal and spontaneous process generated by the learner that is created through connection and empathy between practitioner and learner (Feldenkrais, 2002; Feldenkrais and Doidge, 2019; Feldenkrais and Kimmey, 1985; Baniel, 2012b). Specific outcomes of each future learning event are not known or structured in advance. This kind of learning differentiates and emerges *from the individual’s current level of skills* in a progression, a layering, that has order to it and that emerges moment by moment from where the learner is. It is transformational in the sense that a 24-month-old child is remarkably different from their 12-month-old self, and even more different from their 6-month-old self; they arrive at these differences through a self-generated process involving a combination of wondering, exploration and trial-and-error (Torres et al., 2016; Thelen, 2001a,b).

It is uncommon for the central role movement plays in learning to be appreciated – particularly regarding the associated processes of differentiation and integration that are required for the emergence not only of movement skills but also of cognition and the breadth of human intelligence (Brincker and Torres, 2018; Trevarthen and Delafield-Butt, 2013; Delafield-Butt and Ciaunica, 2024). There may be other sources of early interference with the organic learning process of the child developing autism, such as heterogeneous biological processes, which are beyond the scope of this paper (Varia et al., 2024). An important early model for the impact of such interference was the proposal by Rubenstein and Merzenich (2003) of an increased excitation-to-inhibition ratio in the brain in autism. Disturbances of this sort could reduce the brain’s “signal-to-noise” ratio, leading to a “noisy brain” (Brincker and Torres, 2013; Torres et al., 2013b) that interferes with the brain’s ability to detect differences, making it harder to differentiate. This deprives the brain (and the child) from sufficient richness of new information and flexibility to enable successful learning.

We therefore propose, based on these frameworks and on clinical experience, that the overarching *model of autism* needs to include *neuro-motor-sensing dysfunction* so that it can make sense of both (1) the commonly observed early onset of atypical motor development (from “clumsy” to severe), which we suggest is a precursor to the later arising symptoms that meet the present criteria for autism, and (2) the significant positive changes we have consistently observed through the application of the ABMNM and FM approaches with children with autism that often surpass what was considered attainable. We suggest that the way these approaches use movement, connection, and empathy reduces the noise level in the brain and facilitates learning that was not possible before.

### 1.3 History and context

The core interpenetration of movement and learning in the practices we present resonates at the theoretical level with frameworks of enactive embodied cognitive science theorizing initiated by the 1991 book *The Embodied Mind* (Varela et al., 1991), which stated that “cognitive structures emerge from the recurrent sensorimotor patterns that allow action to be perceptually guided” (TEM, p. 173), and has been further elaborated (Di Paolo, 2005; Varela et al., 2017; Ward et al., 2017), including with regard to autism. De Jaegher (2013), for example, reviews research describing differences between ASD and neurotypical individuals regarding perception, movement, embodiment, sense-making, and salience.

We fully agree that the reality of cognition is inseparable from the phenomena of movement and its associated sensations, though we would add proprioception to perception. The work we are describing is grounded not in description and comparison of features in subgroups, even though our body of work also involves accumulations of observations, knowledge, and categories of phenomena. In terms of our practices, we look for and focus on the client’s dynamic uniqueness throughout the session, in the here and now, what we call “going with the system”. We interact with them on *their* “dance floor” - i.e., we join them in *their* reality and capabilities. We provide them with opportunities to experience that moment – and there to allow them to experience variations close to where they are at so they can spontaneously arrive at new configurations, perceptions, understanding, and actions.

The approach of “going with the system” derives from the work of Dr. Moshe Feldenkrais (1904–1984), a mechanical engineer, quantum physicist, and black belt in judo, who also extensively studied biology, anatomy, neuroanatomy, and physiology. Due to a serious sports injury, he launched extensive, subtle movement experimentations on himself, aiming to change old habits of movement. This led to enhanced awareness and organic learning that yielded new ways of moving and led him to regain previously lost function. In parallel he developed a holistic understanding of human learning and potentiality informed by his diverse domains of knowledge and presaged developments in neuroscience of the last half-century.

Both methods presented in this article evolved out of the work of Dr. Feldenkrais [Baniel (who developed ABMNM from FM) studied with Feldenkrais; Almagor (who practices FM) studied with Feldenkrais and Baniel]. Over time, our experiences in FM and ABMNM led each of us to realize that through *empathy* and by *connecting* with the child wherever they *are* in the process – not where we would *like* them to be – immediate, self-generated positive changes occurred in the child, i.e., we were enabling spontaneous organic learning. We find that this contrasts with many conventional therapies where the focus is often on the presenting behaviors and symptoms which are considered “undesirable,” and where the effort is instead to try to directly change or eliminate these undesirable manifestations, mostly through commands and rote repetition. When the autistic child is erroneously perceived as “resistant” to change, or when they plateau in their progress, there is a danger that these challenges will be attributed to their condition, ASD, rather than to the possibility that the intervention itself, and the way it is delivered, may in fact be limiting the child.

We suggest, based on our extensive empirical experience, that for the remarkable potentials of organic learning to be accessed, movement, connection and empathy – as will be described below and which are universally applicable – need to play central roles in any intervention program, and will have particularly great benefits for the child with ASD.

The specific ways in which our interventions take place will be outlined in this article separately for each distinct method through case studies, vignettes and discussion. We will begin with ABMNM, even though it emerged later than FM, because Anat Baniel’s highly granular case study and analysis will, among other things, prepare the ground for Eilat Almagor’s subsequent FM presentation.

In recent years sensitive technologies and research methods have emerged to measure with high precision the dyadic dance that takes place in our interventions and to characterize the subtle changes that occur during the ABMNM^®^ and the FM interventions. It is our intention to utilize these new developments (Thelen and Corbetta, 1994; Whyatt and Torres, 2017; Kalampratsidou and Torres, 2018) to dynamically measure the learning that takes place, to characterize its relationship to changes in the signal-to-noise ratio in the brain as the intervention proceeds, and to richly describe the exploratory nature of the dyadic exchange. We will present here how such practice-based research might be conducted in collaboration with investigators who embrace the notion of the dyadic relationship between practitioner and recipient (Bermperidis et al., 2023; Torres, 2024) as a means for the recipient to become empowered through a greater sense of motor agency and autonomy.

## 2 Anat Baniel Method^®^ NeuroMovement^®^ and the anatomy of connecting

### 2.1 Part one: case study, Jonathan: first session

The following is a first-person description of my (Anat Baniel) first session, 22 min long, with “Jonathan,” (henceforth “J”), a 21-month-old boy with ASD. In what follows, I present some of the what, how, and why of what I did during excerpts of this ABMNM session. A 6-min video excerpt is available for viewing in ABMNM Supplementary Video 1.

#### 2.1.1 Case presentation and discussion

In each of the following sections, an objective description of what was done and what happened is presented in boldface and followed by a non-bolded discussion of the rationale and other considerations.

**J was brought to me after receiving an ASD diagnosis a few days earlier. His parents reported that he did not make eye contact, refused most foods, was non-verbal, and did not respond to his name. He did not play or relate with his twin brother or other children, and frequently hid under furniture.**

J’s set of symptoms is consistent with a diagnosis of ASD.

**I invited both parents to sit down, and I asked the father to have J sit in his lap, facing out, with J’s back leaning against his father’s chest.**

I began the session by intentionally creating an environment where J would feel safe. This is of utmost importance for the child’s brain to be available to learn and change. For J, I was a total stranger in an unfamiliar setting. He had no reason to trust me, and there was plenty in this new situation that might cause him to feel unsafe. Therefore, I had him stay in his father’s lap for the whole session with me. Fear or stress during the intervention shifts the child’s attention from learning to self-preservation. When this happens, there is a risk that the observer will assign the child’s seemingly inappropriate behavior, or limited progress, to their ASD, when it is actually a manifestation of the child’s *subjective experience of discomfort or fear* during the intervention. With ABMNM, our intention is to *generate a process* that reduces the individual’s stress and fear and creates a safe and pleasurable learning experience for the child.

**FIGURE 1:**
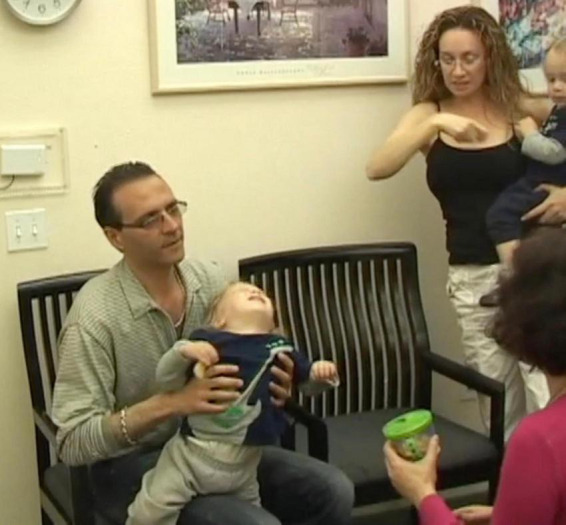
I (Anat) sat facing J. J abruptly and forcefully arched his back, throwing his head way back, then returned to leaning against his dad.

**I (Anat) sat facing J. J abruptly and forcefully arched his back, throwing his head way back, then returned to leaning against his dad (Figure 1). I had observed him doing this arching earlier while his father held J in his arms before we all sat down and started the session.**

While arching was not specifically on the list of possible ASD related symptoms, it was a repetitive behavior, a category which is in the ASD criteria. It was obvious to me that the arching was a manifestation of a disruption in his brain’s functioning, which interfered with J’s ability to pay attention, let alone be present or responsive to any demands placed on him. That was where *he* was at that moment. And it is why I put his arching ahead of all other concerns. It was what I chose to focus on, and where *I chose to join with him in his world.*

**FIGURE 2:**
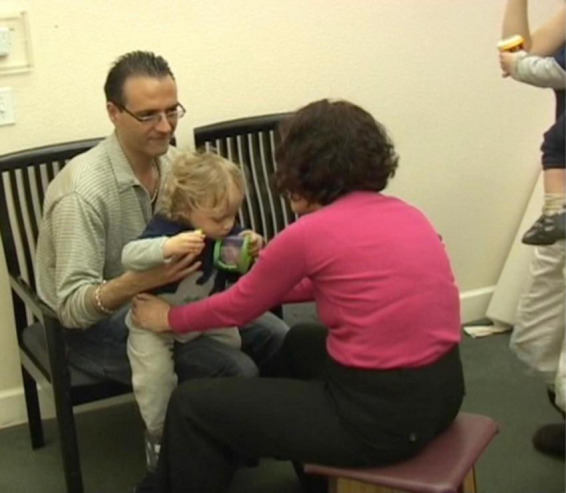
Expecting J to arch again, I gently placed my hands on his pelvis – on his iliac bones – in a way that did not put any weight or pressure on his pelvis.

**Expecting J to arch again, I gently placed my hands on his pelvis – on his iliac bones – in a way that did not put any weight or pressure on his pelvis (Figure 2)**.

My intention was to make clear contact with J without causing him to feel that I was guiding him to move in a particular way, or that I had any expectations of him. My touch was intended only to communicate my presence and for me to connect with J and feel how he was doing his arching. J may or may not have felt or been aware that my hands were on his pelvis. The sensations may have been absorbed into the general “noise” in his brain (which was discussed in section “1.2 Learning”).

The importance of touch, especially for the infant and developing baby, is well recognized. However *not all touch is equal from the point of view of the receiver*. In most conventional movement-related interventions, touch is usually done in a “top down,” way, that is, the goal of the therapist is to get the recipient of their touch to respond in a pre-determined way. In contrast, by touching J fully, without applying weight, or other forces on his pelvis, *I became attuned to him and could begin to receive information from J’s movements through this connection.*

Then I waited.

**As J, once again, began arching abruptly and forcefully throwing his head back, I joined with him in his movement by gently, yet clearly, pressing on his iliac bones, helping the top of his pelvis to roll forward. That is, I connected with J by feeling what he was doing and joining his action**.

The Latin root of the word *connection* is *connexionem*, “*a binding or joining together*.” As I waited with my hands on J’s pelvis, I could *feel* when he began the *involuntary arching* movement again. While I supported what he was already doing, I also exerted just a bit of directed force, *while making sure to not take over his action. This supported the action he was already doing without changing it, and at the same time added sensory input that might facilitate his beginning to notice what he was doing.*

We call this activity of connection “*Going with the system.*” It may seem counter intuitive to “encourage” such involuntary, disruptive action that most likely the child is unaware of. It may seem like we might be doing something that could result in more of the unwanted arching. But on the contrary, in my experience, “going with the system” creates the conditions for the child to *feel themselves* and to *notice what they are doing*. This introduces freedom for the child to either stop what they are doing or do something different. Going *with* the system in this way is different from the common tendency to try to stop or inhibit what we consider to be the “undesirable” action through verbal commands, prompting and, in more extreme situations, exerting physical control.

**Before the third arching, I began using my voice, “singing” in concert with the intensity of his movement. At the end of this arching cycle, he very briefly looked straight into my eyes.**

During J’s third round of arching, after J had already arched and thrown his head back a couple of times, I felt an increasing intensity in his arching movement, so I added my voice, using it rhythmically, with added intensity as his movement intensity grew. This provided J with a second sensory input (auditory) that could be associated with the kinesthetic one; this created an opportunity (though not a certainty) for J to “wake up” and *to notice* something distinct within himself, or outside of himself. *And he did.* At the end of this third cycle, as he was bringing his head back up, he briefly looked straight at me, then quickly averted his eyes. *He noticed me and I noticed that he noticed me. This is something that he hadn’t done before – a real change*.

Having my hands on J’s pelvis and supporting it in rolling forward is not a “technique” which is supposed to get specific outcomes, such as getting a child with ASD to start making eye contact. Rather, I used it as an opportunity to connect with J, and to accentuate the movement of the pelvis in relation to what he was doing, thereby increasing the *likelihood* that he would *feel* his pelvis as part of his action.

I propose that I became a reliable part of J’s re-afferent input from his arching movement because of the way I stepped in—i.e., how I participated helped to *bring the sensations from his movements to the foreground*, making them less chaotic, and more distinguishable from the background “noise” that is always there (Brincker and Torres, 2013; Torres et al., 2013a). This opened the opportunity for J to *feel* himself and notice his own body more clearly. This emerging new ability *spontaneously* extended to his visual perception as he began to notice, perceive, and pay attention to his surroundings.

**FIGURE 3:**
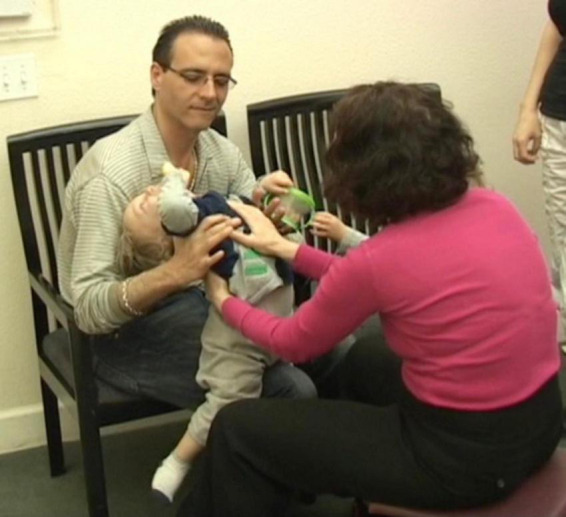
He then went into his fourth cycle of arching while I removed my hands from his pelvis and placed one hand on J’s back, supporting it, and my other hand on his sternum (chest).

**He then went into his fourth cycle of arching while I removed my hands from his pelvis and placed one hand on J’s back, supporting it, and my other hand on his sternum (chest) (Figure 3)**. **I once again joined him in his arching movement by gently helping the sternum move up to facilitate the movement of his head backwards while at the same time supporting his back**.

To further facilitate and amplify J’s feeling and sensing of his arching movement, I moved my hands away from his pelvis and placed them on his back and chest. This is a variation, something new, which gives another opportunity for J to feel and notice himself. I again joined him in his arching action by supporting his back and helping his chest move up as these are also integral parts of the arching movement.

**In the process of arching, when J arched himself so far that he could be looking at his father’s face, I told J, “Look at Daddy.” As he began returning from the arching position, I told him, “Come back”.**

By telling J to “Look at daddy,” I offered a *context* for J’s *involuntary* arching movement to open the possibility for it to become a *voluntary, intentional* movement. I did not know if J understood me. Even if he did not, the questioning tone of my voice might guide him to try to *make sense* of the communication.

**While J’s head stayed way back for a while, I began to gently move his torso in slightly changing directions, thus creating variations in relation to his arching position. Very quickly the ease and mobility with which his torso moved greatly improved, and his pelvis spontaneously began moving in perfect coordination with the movement of his torso. Clear, harmonious, dynamic relationships between the different moving parts of his body emerged in a way that required minimum effort and produced maximum efficiency. J brought his head forward and leaned into his dad’s chest; from then on, J’s involuntary arching stopped and did not appear again**.

Spontaneous variability in movement is central to the acquisition of new movement skills, and to all learning for newborns, babies, and children. J’s forceful involuntary arching was stereotypical with little variability, which is common in children on the spectrum. As J stayed for an extended time in the arched position, I did not try to bring his head back to place. I did not try to correct or inhibit his involuntary arching. Instead, *I connected to him as he was* at *that moment* by placing my hands on his ribs and beginning to introduce movement *through many small variations*. Variations are differences; they provide novelty which opens opportunities for the brain to notice, perceive, and create new neural connections, i.e., differentiation.

**Next I moved my hands to his legs**.

Rather than continue to repetitively move J’s torso in an attempt to “groove in” the recent changes, I got hold of his legs and began moving them, which introduced additional novelty. This also provided him with the opportunity to connect the movements of his legs to the previous movements of his pelvis and torso.

**I got hold under J’s right leg and foot and immediately felt the stiffness in his right ankle**.

I have observed that many of the children on the autism spectrum I have worked with who can walk, have stiff ankles and their backs are rigid, i.e., *under-differentiated*. They move their backs more like a “block.” I propose that this may be due to insufficient variability in the early spontaneous random movements of the child that continues as they develop. Movement in the gravitational field requires constant adjustments of the relationships between the head, back and pelvis to the legs and feet in order to not stumble and to maintain balance while walking. When the back is limited in its ability to change its organization in response to changing demands, the ankles become rigid in an effort to avoid falling.

**As I began moving J’s right leg, I noticed that J was looking at me, then at his leg where I was holding his foot, then back at me with new and very great focus and interest**.

This was a spontaneous new capacity for J, to *notice* and *recognize* his foot; to *feel* and *notice* that something is going on *over there*; to recognize I was there; and to make the connection between his experience and me being there.

J was paying attention and making sense of his experience. *I cannot emphasize enough how important such moments can be!* I immediately shifted my focus from moving his legs, to join J in what he was interested in, which was his foot. I then introduced a host of variations.

**I pointed J’s right foot toward him, touching it, and telling him: “This is your foot.” Then I lifted his other leg and pointed to his other foot telling him: “you have another one of those here.” He followed what I was pointing to with his eyes, and intermittently also looked at me. I lifted his right leg again and began moving the foot and ankle gently in different directions. When I sensed he was about to go into another arching, I uttered clearly “ah, ah, ah,” which shifted his attention back to his foot, and his arching did not take place**.

**FIGURE 4:**
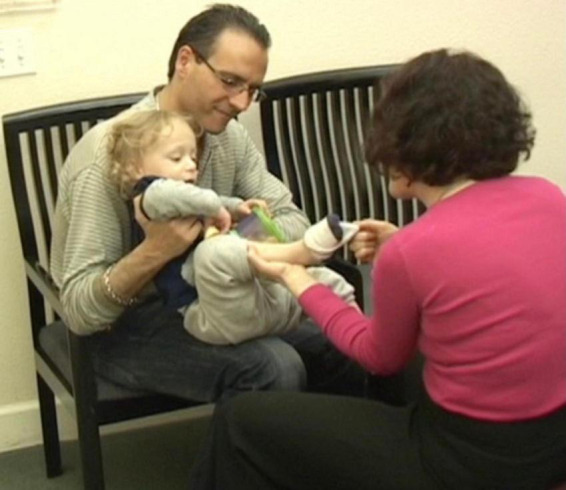
I then said “this is your foot, let me show it to you, and began taking the sock off, slowing way down and exaggerating the process.

**I then said “this is your foot, let me show it to you, and began taking the sock off, slowing way down and exaggerating the process”** (Figure 4).

**FIGURE 5:**
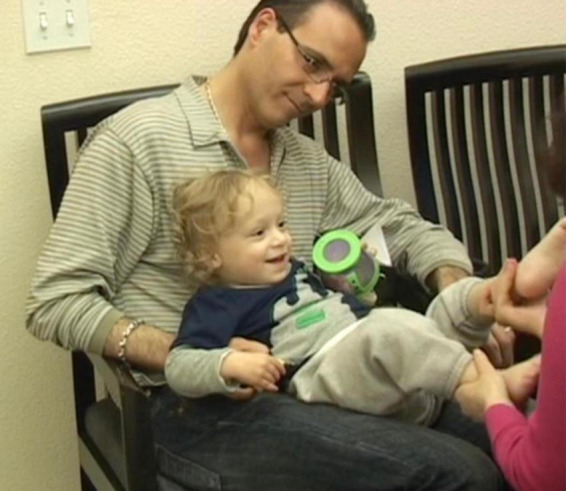
From this point, J was alternating between looking straight at me for long periods of time, to looking at the leg I was moving. He was fully and continuously attentive, indicating that he is aware, and taking in the experience.

**I blew on his bare foot. J smiled and looked at me intensely. I then did the same with the other foot and sock while narrating what I was doing. From this point, J was alternating between looking straight at me for long periods of time, to looking at the leg I was moving. He was fully and continuously attentive, indicating that he was aware, and was taking in the experience (Figure 5).**

**J was noticing! He was making eye contact! He was beginning to make sense of what was happening to him and of what he was doing. By shifting my focus to J’s focus, I joined him and thus maintained my connection with him. If I were to continue with what I was doing with J before I noticed him noticing his foot, we would have lost our shared space (Stern, 2010) and I would have been interfering with his spontaneous self-generated learning process. Instead, the gentle variations I provided in relation to his current interest further amplified the distinctions in what he was noticing, which facilitated for him to keep applying his attention to it. When I began blowing air on his feet, J looked at me intensely and smiled each time I did it. J was connecting to himself and to me as I was connecting to him. It is important to note the significance of such moments. J was exhibiting self-awareness and awareness of his environment. It has been my observation that this kind of change in the functioning of the brain can continue to be built upon, opening up a world of possibilities**.

**FIGURE 6:**
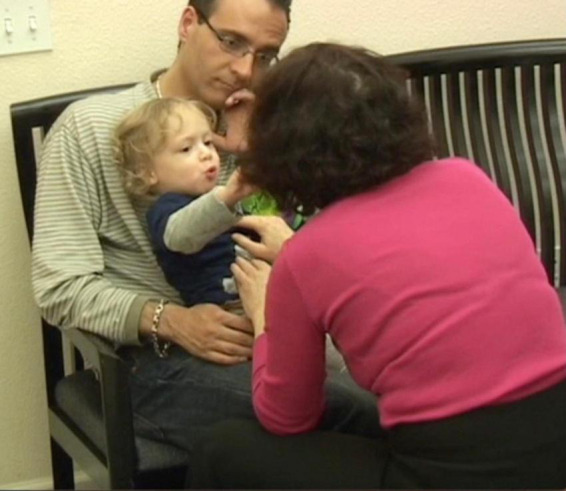
During the session J briefly tried feeding me the apple he was holding.

**During the session J briefly tried feeding me the apple he was holding (Figure 6)**.

**FIGURE 7:**
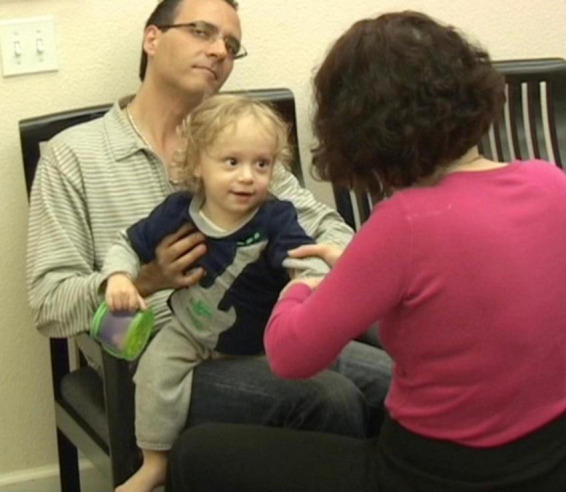
He giggled with delight when I was “shaking” his arm; he responded with “ah” for “yes” when I asked him if he had like me to do it again, and he looked at me in anticipation, expecting me to do it.

**He giggled with delight when I was “shaking” his arm; he responded with “ah” for “yes” when I asked him if he had like me to do it again, and he looked at me in anticipation, expecting me to do it (Figure 7)**.

**Toward the end of the session, J noticed my hands and gently began touching and moving my fingers in different directions, exploring them with great interest**.

During the session, as J’s involuntary repetitive arching dissipated and disappeared, his movements became progressively more fluid and harmonious. Concurrently, he spontaneously began noticing more and paying attention with increasing duration, while his eye contact and directed gaze at what was happening with him and around him spontaneously appeared and increased in conjunction with the motor progression. In tandem with all of the above, J emerged into being relational with me. Movement done in the ways I have described in the sessions with J generated positive, whole system shifts in J’s motor, perceptual, cognitive, emotional, and relational domains. Based on my experience, with many children, I propose that J’s changes point to two things: (1) movement can serve as a portal for significant gains in functioning of the child with ASD, and (2) the changes are multidimensional—not just about the movement itself, but also with broader ramifications that emerge from improvements in the brain’s ability to organize itself and make sense of the incoming stimulation.

**The first ABMNM session was 22 min long. J got six additional sessions during the subsequent 2 weeks from my colleagues. The duration of each session is normally between 30 and 45 min. The mother reported the following changes after these 2 weeks:**

“[Before we started ABMNM] Jonathan did not have (make) contact with his eyes. When we called him, he never came. He didn’t respond to us. And now he’s a completely different kid and he eats better, he sleeps better. He responds to what we have to say. And when we call him, he comes. Great eye contact. He can play with other kids, which is something we didn’t get to have before. So big improvements and talking a lot more, expressing himself. Words! And one more thing, he can fight with his brother, stand up for himself!”

J continued to receive 7–15 sessions every 3 months for a year and a half, then on a need-only basis–when any of the ASD symptoms seemed to reappear–until he turned 6 years old. By that time J was fully verbal and fully integrated into the classroom. Currently his mother describes him as a typical teenager.

It is important to note that every child with ASD is unique and has their own trajectory of progress depending on the age we start working with them, what interventions they had before, and their unique presentation of ASD. A commonality in progress we observe in the children we work with is their gain in agency.

### 2.2 Part two: commentary on case study of Jonathan

In the Commentary below we elaborate further on our approach to working with children on the spectrum.

#### 2.2.1 Connection

In ABMNM we use the term “*connection*” to describe not just a caring feeling for the child, and not just the literal sense of connection through touch, though both are important ingredients. As we use the word here, “connection” refers to the practitioner’s ability to perceive and feel the child’s *own subjective* experience, motivations, intentions, and current capabilities, i.e., *their reality.* The practitioner is highly trained to continuously gather such information through all of their senses, and to a large extent from the child’s movement and its qualities. The practitioner uses this relational information as the key resource from which the intervention unfolds.

Once the practitioner has made this connection with the child, they use their highly trained capabilities to feel and perceive the finest variations in the child’s movements and expressions, so that they (the practitioner) can match the child through their own actions. In this way, they create a loop, a dance, a shared space (Stern, 2010) with the child that is within the child’s own reality. The practitioner *chooses* to initially join and follow the child, so the child is the leader, and the practitioner is the follower. Then the session flows, with the practitioner becoming the leader of the session by guiding the child’s movement into *very subtle variations* of what the child is doing at that moment. *The practitioner is not “correcting” the child’s movements*. The child feels these variations as part of themselves, and as their own spontaneous explorations, *which is the process of organic learning*. These variations lead to the child’s perception of differences and new neural differentiations, some of which may get incorporated into what the child will do next. (Jenkins et al., 1990; Nudo et al., 1996; Merzenich and deCharms, 1996). As the practitioner perceives these changes, or the lack thereof in the child, and again follows the child, they may then introduce new subtle variations emerging from what the child is doing at that moment. This dance continues until the session is over.

This process mimics and recreates the early—what some call “purposeless”—movements of infants, and it fosters the spontaneous, self-generated, exploratory nature of children’s development and learning. It is the essence of what Feldenkrais named “organic” learning. In the loop, the practitioner provides reafferent stimulation that greatly augments the signal. This opens a door of opportunity for the child to perceive differences and for their brain to have new information to work with. Just as with TD children, once the child with ASD experiences – through the intervention – spontaneous, exploratory movements, they begin to self-discover; as the “noise” level in their brain reduces, they are more able to make sense of their experience, with concurrent growing sense of agency. The learning and changes that occurred in the session with J were initiated by J, *felt* by J, and done by him.

#### 2.2.2 Fixing

“*Reality is made up of relationships between objects rather than the objects themselves.*” (Rovelli, 2021).

From the moment a child is born, their bodies are subject to the laws of Newtonian physics. Early on they begin to associate their movements with specific outcomes. Infants knead the mother’s breast to enhance the flow of milk as they nurse. They hit hanging objects on their “baby gym” to get them to move. They hit stacked blocks to get them to tumble. As adults, when an object breaks or malfunctions, we can try to fix it. We can glue, nail, saw, shave, replace parts, stitch, etc. All are ways to interact with and repair physical objects. When a child has ASD, we may attempt to “repair” or “fix” the symptoms that they exhibit the same way we would approach fixing inanimate objects. Yet, while the human body is a mechanical structure that is bound by the laws of Newtonian physics, what changes everything is that it has a brain. The brain is what generates, organizes and forms all human actions. The brain is not a mechanical system; it is an information system that works by different “rules” - by probabilities of outcomes, not certainties (Varela et al., 2017). When the child with ASD is unable to perform desired actions, or performs them poorly, and when undesired behaviors are present, the “fixing” approach fails to provide what the brain needs in order to change and learn.

#### 2.2.3 The “noisy” brain

The brain is an extremely dynamic, complex, self-organizing system that forms itself through its experiences. It is a massive “*information-generating and processing machine*” (Singer, 2009). For learning to occur, there needs to be sensory stimulation. However, stimulation alone is not enough. By itself sensory stimulation is just “noise.” For example, imagine you are in a very loud crowd and a friend is talking to you. No matter how hard you try, you cannot discern what they are saying. Your brain is unable to perceive the difference between your friend’s voice and the background noise of the crowd. “Noise can be defined as any kind of sensed phenomenon or change that cannot be interpreted as a signal.” (Brincker and Torres, 2013). Stimulation turns into information when your brain detects and perceives a foreground stimulation that is distinct from the always present background noise, what is called in science and in engineering, “signal (your friend’s voice) to noise (the crowd) ratio.”

*The process of the perception of differences* and the creation of new connections and patterns, *i.e., learning*, can be seen as having three main steps:

1. *Discrimination*: noticing something.
2. *Differentiation*: the creation of new neural connections in response to discrimination (Baniel, 2012b; see Supplementary File 2: ABMNM- “Your Child’s Amazing Brain”).
3. *Integration*: the spontaneous creation by the brain of new neural networks of movement, thought, feeling, and behavior. “*The job of the brain is to put order in the disorder and make sense out of the non-sense.*” (Feldenkrais, 1981a).

Through my experiences working with children with ASD, I gradually realized that they experience the world like a soup, a blur, which I refer to as a “noisy brain,” which interferes with the process of organic learning. “*Expertise (or the lack thereof) in dart throwing, driving or any other muscle-involved action, is a sign of how well someone has learned to manage the noise.*” (Wolpert, 1992). I propose that the challenges experienced by the child on the spectrum have nothing to do with their “intelligence.” Where they need help is in enhancing their brain’s ability to perceive differences. The “noisy brain” can be witnessed early in development on the motor level (Torres et al., 2013a), prior to discernable social or behavioral features.

When an external and repeated demand is placed on the child with ASD to perform in a way that they are unable to do, this demand is being placed on a brain that is challenged in its ability to perceive the necessary differences, i.e., the information to be able to make sense of the demand placed on them so they can learn. This promotes the “grooving in” of their limitation even deeper in the brain and further degrades their ability to learn.

#### 2.2.4 Movement

“*Nothing happens until something moves.*” Albert Einstein.

Human beings are born completely dependent. At birth they have a brain that needs to grow and form roughly an additional 78% to reach its adult size (Dekaban and Sadowsky, 1978). Movement begins *in utero*; there and at birth the infant has no voluntary control over their movements. They initially move mainly through reflex and random movements. Once born, these movements are performed in the gravitational field, where the infant *feels* the weight of their limbs, head, and the rest of their body. The pressures on their body are now distributed unevenly, depending on the position they are in in relation to the gravitational field; and these pressures keep changing as the child moves or is being moved. Every movement of the body produces continuous change that generates a flow of associated sensations. *These sensations call the child to attend to what they are feeling.*

An additional constant companion to the movement is the interaction of the movement with the environment. Those interactions generate outcomes that also call on the child to attend to what they are experiencing. For example, when an infant lies on their back and randomly kicks the floor, they feel an unexpected pressure on the heel and a push upward through the spine toward the head. This reafferent experience drives the creation of multiple connections between the heel and the brain – what is called *mapping* of the body to the brain. As mentioned earlier, when the nursing infant is held on their side with one of their arms free to move, that arm, sooner or later, will unintentionally lift up, then “land” on the breast and press on it. This pressure, when sufficient, will increase the flow of milk. When this happens, the baby gets an unintended outcome to a random, unintended movement. When enough variations of the lifting movement of the arm and dropping it toward the breast have occurred, some of which were “successful” in enhancing the flow of milk, and others not, the child’s brain fills in the *mapping of the arm in relationship to the outcome.* With it, the brain hones in, decreasing the randomness of the movement and increasing the probability of success, until it becomes an intentional action on the part of the child.

This process consists of continually increasing complexity, refinement, and degrees of freedom through which new skills arise as the brain continues to self-organize. We suggest that emerging from this process are also awareness, thinking and cognition. The brain of the ASD child also self-organizes. The question is, at what level and quality of organization will it create? We further suggest that when we shift our focus from trying to “fix” the ASD symptoms by attempting to get the child to do what they cannot, to *connecting* with the child where they *are*, the child can get better outcomes and the brain itself can get better at learning – i.e., it *learns how to learn*. They will “improve their abilities and build a better, stronger brain” (Merzenich, 2009).

### 2.3 Part three: The Nine Essentials: mapping the way from Fixing to Connecting

In the ABMNM methodology we have defined what we call the nine essentials. We propose that when we bring the nine essentials to an intervention with a child who has ASD, a particular *connection* is created that greatly potentiates that child’s learning. We can bring the Essentials to the intervention process with children on the spectrum in a disciplined and intentional way, to generate what we have discussed as *organic learning*. Organic learning is naturally present for TD children, particularly in the early years of their lives, when the most potent and greatest amount of learning takes place. The nine essentials provide guidelines that help us over time to get better and better at generating the conditions where the child with ASD can learn and change. The nine essentials are particularly accessible for practitioners because each essential has its roots in experiences familiar to us all. Current neuroscience research supports our contention that the *Essentials* are drivers of neuroplasticity and positive brain change (Merzenich, 2011).

The nine essentials, briefly described here, are more fully described in Supplementary File 3: ABMNM-“The Nine Essentials”.

#### 2.3.1 Movement with attention to the feeling of self

Movement is present in all action and behavior. However, movement by itself is not enough. Learning and positive change require that the child bring their attention to the *sensations* associated with their movements, to what they *feel*. Movement with attention is a way of communicating with the child that upgrades the functioning of their brain.

#### 2.3.2 Slow

Fast, we can only do what we already know. To learn and master new skills, and overcome limitations, the first thing to do is to slow way down. Slow helps the brain to notice differences, to sense, and to feel. It requires that the practitioner, therapist, teacher, or parent slow themselves down, so they can feel and recognize more of what is happening in themselves and in the child. Slow allows true connection.

#### 2.3.3 Subtlety

A powerful way to enhance the ability of the child’s brain to perceive differences is to reduce the force, or intensity, of stimulation with which we interact with the child. When we reduce intensity in our interactions with children, we are also training ourselves to shift from fixing to connecting.

#### 2.3.4 Variations

Variation is a necessary ingredient for learning. Variations can be generated by varying speed, size, direction, content and intensity. They can come in the form of play, “making mistakes,” creativity, or exploration. They make learning possible and *fun*.

#### 2.3.5 Enthusiasm

Enthusiasm is self−generated. It helps to bring the experience to the foreground where it is more likely to be noticed and learned. However, this is not about clapping, cheering, or telling the child how good they are. It is an *internal*, quiet and intentional process where one *chooses* to feel delighted about seemingly small changes in the child.

#### 2.3.6 Flexible goals

Our society places great value on goal setting, including the expectation that goals need to be achieved within a particular time frame. To follow the dictates of rigidly preset goals, the practitioner disconnects from the child’s experience, from reality and from their own internal experience. This severs the connection and interferes with the child’s ability to learn.

#### 2.3.7 The learning switch

At any given moment the brain is either in a *learning* mode – when the learning “switch” is on – or it is not. You know that the “switch” is on when the child spontaneously pays attention, shows interest, and initiates their own involvement.

#### 2.3.8 Imagination and dreams

In ABMNM we often utilize imagination as part of the intervention. Imagination, like all other skills, can be developed and enhanced, contributing to the child’s ability to learn.

#### 2.3.9 Awareness

When working with infants as young as a few days old, I noticed that the moment I connect with them in the ways described in this article they quickly stop doing what they were doing – i.e., crying, twitching, flailing – and become attentive to what they are feeling. By noticing and joining with the awareness of the child, a loop – a dance, if you will, is created that results in a vibrant process of learning and change.

## 3 The Feldenkrais method

### 3.1 Orientation to the Feldenkrais method

The FM is a learning process that takes place in the course of gentle movement lessons. It is aimed at enabling individuals to improve their abilities by developing their awareness of the self and their way of moving. The method uses movement as a natural learning process, similar to the way that infants come to know themselves and the world around them. Infants learn to roll over and crawl, to identify themselves and their limbs as they become familiar with their human and physical environment, and to stand and walk, all in the process of practice and play, errors, falling, and succeeding. Gradually, based on their senses and their rich collection of experiences, they choose the actions that serve them with the greatest efficiency and pleasure.

These learned actions develop others, and the nature of one action depends on the preceding one. For example, the way one crawls is influenced by how one rolls over, and the way of standing depends on how one crawls.

Infants develop habits based on exploration, in what Moshe Feldenkrais called “organic learning.” He created the Awareness Through Movement – ATM lessons in a way that promotes such organic learning. Thus, like infants, for adults, too, organic explorational learning becomes a tool for improvement. Feldenkrais defined improvement as something that emerges from one’s own abilities, through one’s own agency, where new information resonates with existing neural patterns (Edelman and Tononi, 2000), rendering a system more complex and more refined, in terms of possibilities and accuracy, than it was.

Teachers of the FM are trained by practicing and experiencing being students of these lessons themselves, either by ATM lessons guided verbally or by Functional Integration (FI) lessons guided by practitioner touch and manipulations. Feeling the human joy and satisfaction of being guided to promote their agency enables them to sense and connect to the others’ personal way of being and moving.

We work in the same way with all students – children with special needs, people experiencing pain, or highly skilled professionals who want to do even better. In every case, our connection through words, touch, and movement enables the student to be and to begin work from any starting point. In other words, they start from a place where their nervous system is well connected to their feelings, movement, senses, perceptions, and experiences. As a result, when they learn a new movement, it is integrated with what they could do before that, which is the organic process in which the nervous system evolves.

**FIGURE 8:**
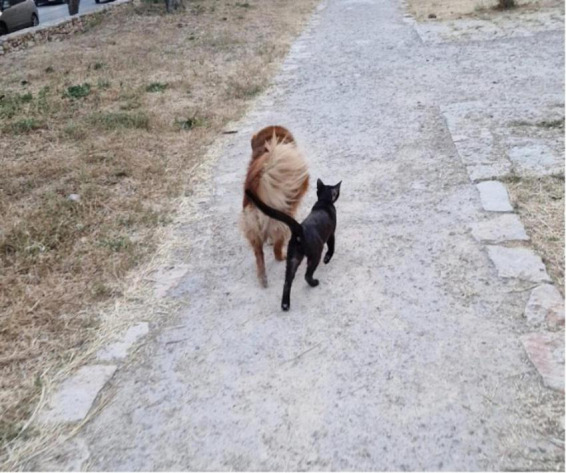
Skillful empathy “Can two walk together, except they be agreed?” Amos 3:3 King James Version, Holy Bible.

We assume that in these dyadic FI Feldenkrais lessons, the two nervous systems – that of the teacher and that of the student – act as one entity. A similar sensitive empathic skillful interaction happens naturally between animals, as in the Figure 8. If you look closely at the photograph, you will see an amazing synchrony of the legs and not just the tails. It is the teacher’s trained awareness and flexibility that allow this to happen, moment by moment. Awareness of the Feldenkrais teacher induces changes in neural resting state activity in the student’s brain (Verrel et al., 2015). A third, omnipresent element involved in the process of learning through movement is the force of gravity.

As Elizabeth Torres put it, whether in FI or ATM modes, “There is another person who guides the process through voice and touch/pressure/kinesthetic senses. There is a closed feedback loop between the body-brain of both agents in the dyad. I can see two feedback loop scenarios; one is in the stand-alone mode of the person’s body sensing back its own self-generated motions. The other is the dyadic one, which contributes the guidance of the partnering agent.”

It is in this sense that one should see Feldenkrais as a very empowering method. It gives people an opportunity to self-discover their agency through this exploratory experience and to improve their bodily awareness through the spontaneous motions that are otherwise largely beyond awareness. Feldenkrais allows the awareness of these motions, akin to when we were infants.

“Organic learning begins in the womb and continues during the whole of the individual’s period of physical growth … other forms of learning directed by teachers take place in schools, universities and colleges” (Feldenkrais, 1981b, p. 29).

### 3.2 Learning through approximations

The natural learning processes take place through approximations. For example, skilled basketball players will get the ball into the basket most of the times they throw it. This ability is developed by numerous practice throws which only come near to the basket. Such approximations are not undesirable errors; rather, they serve as an important series of different experiences with different “orchestrations” of the senses, organization of the movements of limbs, and taking aim. The richness of these orchestrations enables improvement in getting the ball into a given basket, but the practice also enables making the basket under different conditions, from different positions and distances, or using a different ball or even a different object. The ability to apply the learning of one ability to learning other abilities is founded on approximations. In neonates and infants, movements are made almost randomly in different variations, even before there is a goal of succeeding or getting somewhere.

### 3.3 Case studies

In the following, two cases are presented to demonstrate the special teacher–student dyad in a Feldenkrais lesson, and the learning that results from it.

#### 3.3.1 A Lesson with Gabriel

Gabriel was born prematurely and was diagnosed with cerebral palsy. Josipa Stipetić Irha, a Feldenkrais practitioner, has been working with him since he was 3 months old. In that time, he has learned to reach, roll, crawl, change from sitting to crawling, and then pull himself up to stand while leaning on the wall.

**FIGURE 9:**
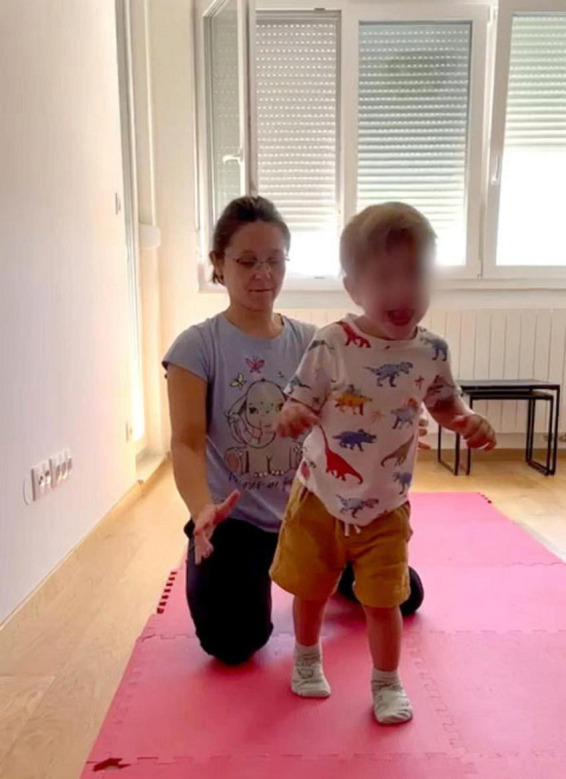
Gabriel walking before the sitting ATM. See short Video 2.

At the age of 2 years and 10 months he took his first steps independently (see Figure 9 and link to Video 2 in Supplementary File 6). The teacher walked in back of him, holding her hands on either side of him and providing support when he seemed to lose his balance, to help him regain stability. The child happily toddled across his room, but it was clear that he was not in full control of the speed and direction of his movement. At a consultation online meeting between Josipa and myself, we looked for conditions in which the child could experience and become aware of regaining stability.

**FIGURE 10:**
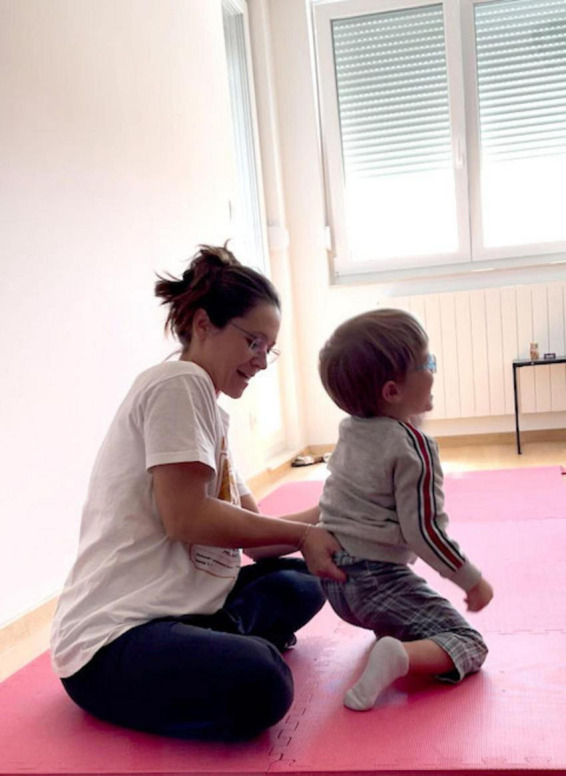
Gabriel in sitting, Lifting and dropping his pelvis, he senses the interaction with gravity. First being guided by the teacher, then gradually becomes active in this exploration. See short Video 3.

We decided to work with him while he was sitting on the floor. The teacher sat behind him, holding his buttock, lifting it a little, and letting it fall back to the floor. She continued with variations of this movement, always lifting the pelvis but with small variations, thus changing the way he sat and the points he leaned on (see Figure 10 and link to Video 3 in Supplementary File 6).

It is clear that both the child and the teacher responded and adjusted their posture according to the lifting of the pelvis. We discern the three entities – the child, the teacher, and gravity – working harmoniously. The teacher was active in lifting the pelvis but allowed the child to experience the fall on his own and sense his regaining of stability.

Long before they stand or walk, infants fall frequently and in many different ways. This experience is essential for acquiring and adapting to an upright position.

It is important to consider standing as a dynamic state, in which there are constant oscillations. The body deviates from the center and then returns to the center. This is a steady state. It is similar to the state of equilibrium, but it requires the investment of energy each time it corrects itself back to the center. The deviations from the center are mostly caused by gravity; they are like small falls, and the return to the center is like getting up from a fall.

Thus, the minimal oscillations that occur when standing are a refinement of falling and getting up. It would be impossible to stand upright without the knowledge and experience of falling and getting up in primary postures such as sitting, crawling and, even earlier, postures like lying. In lying, when the baby lift parts of their body off of the floor and these parts fall back to the floor, the experience is a combination of sensing the pull of gravity but also of the interplay between parts that lean more and parts that lift more. This interdependency between one’s parts in the presence of and due to gravity seems to be an important part of the development of a sense of self and of agency. The level of interdependency between one’s parts increases ontogenetically, as the postures entail lifting more parts off the floor, and eventually standing upright. In standing upright, the neural organization must include readiness for the possibility of losing balance, responding either by falling safely or by regaining stability and returning to the center. Based on this theoretical line of thought, Josipa and I chose a lesson for Gabriel to experience falling, but in a less demanding state, when he was already on the floor (as shown in link to Video 3 in Supplementary File 6).

**FIGURE 11:**
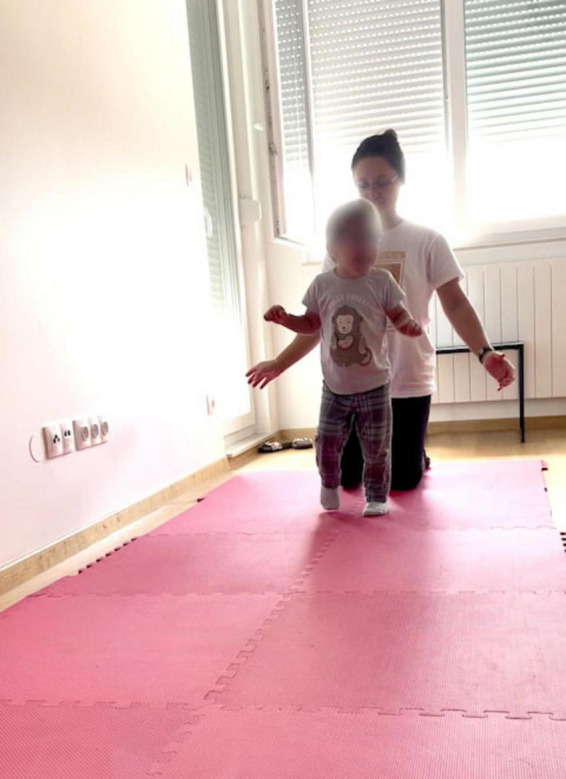
Gabriel, walking after doing the sitting lifting/dropping pelvis. Walking is smoother with less wobbling and better dynamic regaining of stability in each step. See short Video 4.

At the end of the lesson, the child seemed to be more in control and able to walk more slowly, stopping on the way and looking around, allowing his arms to hang down (see Figure 11 and link to Video 4 in Supplementary File 6). This indicates that he was walking with greater degrees of freedom, reflected in flexibility in movement, synergetic use of more joints and muscles, and greater differentiation. Specifically, while standing on the R foot he places his pelvis better on the hip joint to support the spine and the head. As a result, he was able to look sideways while he was walking forward. This, in turn, can be expected to enable other, more complex movements to emerge. Having more degrees of freedom facilitates regaining stability, which is essential for walking.

This example shows how the FM uses basic positions and movements already known to the child. In performing them, the child recognizes and explores what they are able to do, and this can be expected to enable them to apply it to other actions, as well.

#### 3.3.2 A lesson with Yochai

**FIGURE 12:**
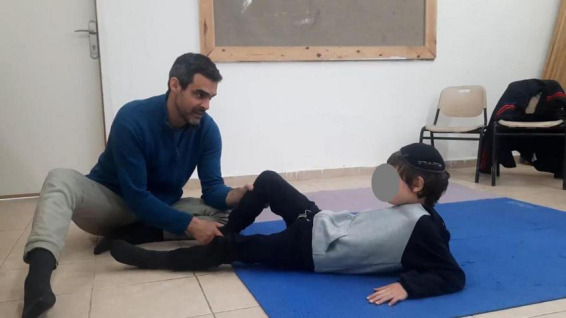
Lesson with Yochai. The FM starts a learning process with what the student already knows. See Supplementary Video 4 which shows the entire lesson.

The video recording of the lesson with Yochai illustrates the strategies of being with the child according to the FM (see Figure 12 and link to Video 5 in Supplementary File 6).

Yochai is a child with ASD. At the time when the described lesson took place, he rarely spoke at school, and never at his own initiative. He would sit for long periods almost without moving. However, at home with his mother, his linguistic and motor abilities seemed to be more manifest.

In the FM, learning starts with what the students already knows. The teacher began the lesson by introducing the basic movement of stamping one’s feet and then proceeded to a movement of opening and closing the legs, which he knew was familiar to Yochai. With both movements, he offered variations that might connect and relate to other movements as the lesson unfolded. For example, he began with stretching the legs and then bending and stamping, which developed into opening and stretching the legs (0:00–0:44), and later, holding the foot with a hand and stretching the leg (13:39). Similarly, opening and closing the knees (1:10) developed into tilting the knees from side to side (1:30, 2:15) while shifting their weight and later on, standing on his hands and knees (4:55).

Feldenkrais teachers only offer a movement but never force it. They are attentive to the students’ responsiveness and interest and respect each student’s autonomy. In the lesson presented here, after few movements of stretching and bending the legs, Yochai held back his legs; the teacher noticed this and immediately stopped (0:26).

By respecting the student’s autonomy, the teacher encourages their sense of agency and creativity. The student becomes involved in leading the lesson and offers their own variations. Thus, after stopping the stretching and bending of the legs, Yochai started opening and closing his legs.

In order to connect with a student in this way, the teacher must be involved in the movement with their entire body. This enables them to be more receptive to slight changes in the dynamic of the student’s movement and better transmit their own intentions to the student. This might be called “skillful empathy” (Figure 8). In this state of mind, everything becomes part of the lesson, which becomes a total experience; nothing is considered an interruption. If the student’s attention is distracted, the teacher follows the student’s new focus of interest. For example, when Yochai suddenly lost interest in what they were doing and focused on his plastic dinosaur, the teacher did not try to draw his attention back, but rather asked him about the dinosaur (3:26). Similarly, when Yochai was bothered by a loose sock, the teacher helped him straighten it out (12:30). Other aspects of this totality are the use of different modalities like sound and voice, locations and orientations, images (e.g., walking like a cat, 5:45), and more. All of these constitute the context of the lesson.

The complexity of the lesson and of the two systems make it a dynamic process in which the dyad behaves as one system. The teacher does not lead it exclusively. Instead, both the teacher and the student lead it. In fact, it is more accurate to say that the process leads itself spontaneously. There is a flow of occurrences and relations. Some moments are intensive and clear; others seem vague or meaningless. All of them are equally important parts of the process.

As in other dynamic processes, phase shifts might occur. The lesson can lead to unpredictable outcomes. The teacher must remain open and ready for such surprises, and recognize such shifts. They might start with small variations that, once recognized and made available, can lead to new possibilities. Such a shift occurred at the end of the lesson with Yochai, when he exchanged roles and started moving the teacher’s legs, first with his own legs, then adding the use of his voice to give verbal orders (for the first time during a lesson), and eventually using only his voice (27:20). The new abilities emerged spontaneously out of the whole process.

### 3.4 Summary: being with the child according to the Feldenkrais method

As described in the Introduction and demonstrated in the two lessons described above, key principles and strategies guide the dyadic Feldenkrais lesson with a child. Note that many of the principles outlined here are also manifestations of the nine essentials of the Anat Baniel Method (ABMNM).

1. A Feldenkrais lesson is an exploratory learning process, in which both the teacher and the students learn, with no strict predetermined goal, but rather discovery of achievements and possibilities during the process. These are related to the Flexible Goal essential in ABMNM.
2. The learning starts with what the students already knows.
3. A trained teacher is familiar with many associated movements, enabling them to offer variations and approximations of the movement and envision possible connections to other movements. This corresponds to the Variation Essential in ABMNM.
4. The teacher only offers a movement but never forces it.
5. The respect for the student’s autonomy and the attentiveness of the teacher encourages the sense of agency of the student, and their own creativity.
6. To be able to connect with the student in such a way, the teacher must also be totally involved in the movement, including the way they move, think, and imagine. The goal is one of holistic empathy, or as some call it, embodied empathy.
7. Everything becomes part of the lesson; it becomes a total experience, and nothing is considered an interruption.
8. The complexity of the lesson and of the two systems make it a dynamic process that leads itself spontaneously. There is a flow of occurrences and relations. Nevertheless, the teacher must take responsibility for the quality of attention, the relationship, and leading.
9. Phase shifts might occur, and the lesson might lead to unpredictable outcomes. The teacher must remain open and ready for such surprises.

## 4 Recommendations for research

### 4.1 ABMNM research

An initial research study has already taken place in a Catholic School District in Lloydminster, Canada. The administrative data collected by the schools shows many improvements; this is summarized in Supplementary File 4: ABMNM Research in a document titled *Report from the Director of Education where the ABClassrooms (Anat Baniel Classrooms) program was implemented.* There is sensor data from this study pending analysis in Dr. Torres’ laboratory. A second document in Supplementary File 5: Research titled *Researching a Ground-Breaking Novel Approach to Learning and Education in Schools and in Children with ASD* outlines the background, preliminary outcomes, and technical features of planned work.

### 4.2 Feldenkrais method research

The complexity of a dyadic Feldenkrais lesson, the subtlety of the changes, the learning that takes place, and the exploratory nature of the process pose challenges to efforts to conduct scientific research of the method. Nevertheless, the development of new technologies and research methods now makes such study possible.

Professor Elizabeth Torres of Rutgers University has developed an approach that might guide such research. Together with her colleagues, Torres suggested a method for characterizing movement, based on its moment-to-moment micro fluctuations relative to the empirically estimated mean activity of the person. This innovative approach differs from the traditional perspective, which assumes a theoretical distribution to obtain the theoretical mean and variance of the behavioral processes under consideration. The traditional approach ignores fluctuations that do not fall within two standard deviations from the *a priori* theoretical mean. This “one-size-fits-all” model, which employs set averages of the fluctuations, fails to consider the nuance in the behavioral stream, which, as it turns out, contains important information about the individual’s variations.

Torres’ approach aims to characterize such variability using a truly personalized approach that empirically estimates the continuum of probability distributions that, according to a maximum-likelihood estimation, best fit the data stream generated by a given person. These distributions and their shifts from moment to moment are like a fingerprint; they describe the person’s natural motions and define stochastic parameter ranges that span from memoryless random with high noise-to-signal ratio to Gaussian predictive with high signal-to-noise ratio.

According to Torres, when using this approach it is possible to obtain a personalized statistical signature of variability for each person and thus characterize the degree to which the impending movement’s consequences are predictable. Greater predictability of a movement’s consequences indicates a larger degree of motor control in the individual’s self-generated actions (Torres et al., 2013b). This, in turn, establishes cause and effect of the person’s actions and their consequences – a form of kinesthetic reafference (Bernstein, 1996; von Holst and Mittelstaedt, 1950) that acts as a continuous stream of sensory feedback, aiding the brain in building internal models of action (Kawato and Wolpert, 1998), action ownership (Torres et al., 2013b) and, together with the motor systems’ autonomy, supporting an overall sense of motor agency (Bermperidis et al., 2023; Torres, 2024).

Using motion detector sensors and video recordings, Torres and her colleagues measure what they call micro movements, which are standardized fluctuations of the movement along a given trajectory, and analyze their accumulation from moment to moment, thus tracking their shifts in stochastic signatures over time. In this way they can characterize movement, identify and quantify a motor learning process, and extend the framework from the individual agent to a shared space between two or more agents of a dyad or a social group. These extensions of the work could include, for example, dancers dancing together (Kalampratsidou and Torres, 2018), a child and a clinician interacting during a diagnostic test like the ADOS (Bermperidis et al., 2023; Whyatt and Torres, 2017), or any other scenario in which two agents interact and communicate.

In earlier research, Torres et al. (2013b) showed that children with ASD responded well to a motor-learning task in which the goal was self-discovered through spontaneous naive exploration, but did not respond well to directed motor tasks with explicitly prompted commands. During the exploratory process, they generated a more reliable set of anticipatory motor statistics; this suggests that evoking the sense of agency acted as a noise-cancellation mechanism that enabled a predictive code in their voluntary motions and bridged their mental intent to the physical volition realizing the intent (Torres et al., 2013b).

This research suggests that interventions that are based on spontaneous exploratory learning might be especially effective with some children, and specifically children with ASD. Such learning lies at the heart of the FM; as mentioned earlier, Feldenkrais (1981b) referred to it as organic learning and defined it as a natural ability by which all the basic skills are learned. In dyadic Feldenkrais lessons, the teacher facilitates the exploratory learning process of the student and provides the conditions necessary for organic learning to flourish. Such lessons conducted with children with ASD at Yad Hamore School in Jerusalem seemed to be successful beyond the expectations of the school staff.

### 4.3 Proposed clinical research

Research of Feldenkrais lessons based on Torres’s method might shed light on the process of exploratory (or organic) learning, as well as the unique relationship and communication between a teacher and a student in such a lesson. In addition, such an investigation could inform the development of an alternative approach for therapeutic intervention for children with ASD children. The first goal of such a research project would be to establish measurable parameters, extracted from the sensor data, that would capture in a rigorous, scientific and quantitative way the subjective experiences of important learning events or processes reported by Feldenkrais teachers and students. As a first example, the research will develop such a method based on the sensor data to identify instances of high-quality learning, connection or empathy, which we will refer to as “organic learning moments,” in a measurable and quantitative way.

In such a research project, both the teacher and student will wear motion sensors during the lesson and will be recorded on video. Before the lesson, both will be asked to perform a simple motor task, which will be repeated at the end of the lesson. The lesson will be observed by a trained Feldenkrais teacher who will watch the child and teacher and mark what we will refer to as “organic learning moments” – that is, moments in which there is a high quality of connection or empathy. An analysis would then be performed to test the correlation between the organic learning moments identified by the Feldenkrais teacher observer and the periods of spontaneous exploratory learning or other parameters identified by Torres’ sensor measurement and analysis. Two other important dimensions to address are the dyadic relation between the teacher and the student and the synchronicity between them that is enhanced by the lesson and greatly contributes to its effect. The quantitative identification of such synchronicity was studied by Torres in lessons involving parents and children. Such research will be conducted to verify that it is indeed enhanced during lessons in both methods and also that its presence indeed contributes to “organic learning moments.”

Subjective documentation by both teacher and observer will describe the lesson as they remember it, highlighting significant occurrences such as high-level communication, enhanced learning, or other breakthroughs. These markings will be matched to the timestamps of video recording. The notes of the teacher and the observer will then be compared to and possibly integrated with Torres’s data set.

## 5 Summary, implications, and conclusion

The FM and ABMNM^®^ are based on leveraging neuroplasticity through the utilization of movement, not as “exercise” or externally imposed motor sequences, but as a means for effective, *two-way felt communication* with the recipient and their brain. Through *connecting with the recipient, starting where they are–motorically, emotionally, and cognitively*, we follow their *unique responses, moment-by-moment*, creating a dance-like dyadic process of self-discovery that mimics the spontaneous, organic way TD children play, learn, and grow.

As we observe the limited success of most current ASD interventions, there are two aspects to consider. The first is the way the intervention is done, which we have addressed. The second is the timing of the intervention. Technologies and metrics are emerging to identify autism risk very early in life, and even potentially *in utero*. Large-scale analysis of perinatal auditory brainstem response (ABR) data has been shown to detect high risk of autism from birth (Torres et al., 2023). The presently dominant interventions focused on social, behavioral, and communication challenges cannot be initiated until divergences in those capabilities emerge and can be detected, which occurs far later in the child’s lifespan. The two interventions we have discussed in this article can be applied shortly after birth and during early infancy. This is because these interventions communicate with the infant and their nervous system directly through touch and movement; they neither rely on the use of language nor require the infant or toddler to generate specific actions or functions which no child can do at that age. We propose, based on our experience, that very early intervention using these two approaches will help avoid having the child go down the full cascade of developmental challenges and failures. Our understanding of why this is possible is that these methods potentiate the emergence of a more successful organic learning process for the child.

The research technologies we will deploy and our practice methods share the qualities of flexibility, nuance, and responsiveness to the uniqueness of the individual. As such they can be deployed to study both the effectiveness of the interventions and the accuracy of the theory and assumptions underlying both the FM and the ABMNM.
