# Supplementary material for: From fixing to connecting—developing mutual empathy guided through movement as a novel path for the discovery of better outcomes in autism
Source: Front Integr Neurosci. 2025 Apr 2;18:1489345. doi: 10.3389/fnint.2024.1489345 (PMC12031662; doi:10.3389/fnint.2024.1489345)
Supplement: Supplementary file 1 [file Supplementary_file_1.docx]

Chapter 2

From Fixing to Connecting

There is no surprise more magical than the surprise of being loved. It is God’s finger on man’s shoulder.

—Charles Morgan

A child is born. It is perfection, a miracle. Without knowing any of the details, and often long before the birth, we have begun creating a vision for that child’s future. He will grow up to have a full and satisfying life, independent and whole. Then comes the shock. Something is wrong with my child! Sometimes this moment comes all at once, at birth or even before. At other times the realization unfolds gradually. The diagnosis and cause might be clearly defined, at least in medical terms, or it might remain uncertain.

As the realization that something is wrong sinks in, bringing overwhelming fear, confusion, and deep grieving (sometimes unconscious) and often a disquieting sense of guilt. But the overriding part of the experience is a powerful desire to do anything and everything we can to help our child. We want to help him grow up to be “normal,” to be able to walk, talk, think, and feel and to have an independent, rewarding life. The questions we must ask ourselves are, How do we get there? How do we go about helping the child grow beyond his present limitations?

We must ask ourselves, How do we go about helping the child grow beyond his present limitations?

When we learn that something is wrong, whatever it might be, the natural thing to do is to focus on the limitation, on what the child can’t do, or on what he is doing wrong, and then we try to conquer, stop or otherwise overcome it. We want to solve the problem, and fix the child so he will sit up, talk, read, write, relate to others and reason the way he would if it weren’t for his special needs.

The desire to fix what is broken or malfunctioning is tremendously important and useful. There are times when fixing is exactly the right approach—for example, when doctors need to surgically repair a hole in the heart, when an antibiotic is used to combat an infection, or when a blood transfusion is needed. When they’re necessary and when appropriate treatment is available, those fixes need to be carried out. At the same time it is important to understand that fixing is but one way of approaching problems, one that has great limitations and can at times even be counterproductive. The Nine Essentials presented in these pages are intended to guide us beyond the limits of the fixing approach­, providing new opportunities for the child’s own brain to create the solutions it requires.

Looking More Closely at the Fixing Model

Most of us have an understanding of fixing as repairing what is broken, or restoring the way something is supposed to look, function, or be structured. When we’re talking about a mechanical object, such as a car or home appliance, we generally know what to do: If we can’t fix it ourselves, we hire an expert who does know. When we have notice a flat tire we have the leak repaired; when the car’s engine misfires we have a mechanic replace worn parts and get it running smoothly again. The repair person employs her brain, her experience, and available resources, such as replacement parts, to restore the machine to its original design and functionality.

Unlike the child, a car or other machine can take no active part in the repair process. It has no mind of its own, no self-healing ability, and it doesn’t possess the capacity to learn, grow, and evolve. The car mechanic’s role in replacing parts and tuning up an engine is the essence of the fixing paradigm. And how natural it is to want to use this same paradigm for a child with special needs! We want to replace any missing parts or parts that aren’t working well. We want to find somebody who knows how to adjust this or that so that everything will be working just right.

Unlike the cars or kitchen appliances, your child is not a finished product. Children are living, feeling and experiencing beings, works in progress.

Unlike the cars or kitchen appliances, your child is not a finished product. Children are living, feeling, and experiencing beings; works in progress—growing and evolving; in the process of figuring out; and continuously forming the abilities to move, think, understand themselves, and relate to the world around them. At the center of all this unfolding potential is that most miraculous of all organs, the brain. Regardless of his special needs, your child possesses a brain that must be actively involved in creating new connections and patterns for overcoming the limitations and difficulties he may have. This is what every brain is designed to do. And this is where our hope lies.

At the center of all this unfolding potential of your child is that most miraculous of all organs, the brain.

To make full use of the brain’s remarkable potentials, we need to make a shift in our thinking, away from the fixing paradigm. To be truly helpful, we need to know how to focus our attention on what we can do to awaken and strengthen the capacities of the child’s brain to do its job well. We are not alone in the process of trying to help the child with special needs; our greatest resource and our best partner in helping our child overcome challenges is the child and the powers of his own brain.

Making the Transition from Fixing to Connecting

To be truly helpful to the child with special needs, we need to back off from trying to make the child do what he can’t do. We need to more fully recognize the fundamental role the child’s brain plays in learning and refining new skills. The brain has the ability to figure itself out and, amazingly, to create successful solutions to problems. No matter how much we may want to help, and no matter what our expertise, we are 100 percent dependent on the child’s brain to make the necessary changes.

Through the Nine Essentials we move quickly beyond the limitations of the fixing paradigm to help the child’s brain do its job better, providing the underlying process by which all skills and all learning emerge. The Nine Essentials will help you to get your child’s brain working brilliantly. Yes, you read that right. In the pages ahead you will discover how your child’s brain can evolve to function in remarkable ways despite the very real challenges he is facing.

The Nine Essentials will help you to get your child’s brain working brilliantly.

Whenever we ask the child with special needs to do what he cannot do, that’s the fixing paradigm in action. Let’s say he cannot sit up; we put him sitting up, and we do this again and again, hoping that after repeating this enough times he will somehow get it and do it himself. If he cannot talk, we ask him to imitate certain words or sounds, over and over again, hoping that with enough reiterations his speech deficiencies will be fixed. Sometimes this approach produces the desired results, and sometimes it completely fails. If we instead focus our attention on awakening and strengthening the process by which the child’s brain can create and discover its own solutions, the results that become possible are of a completely different order.

Whenever we ask the child with special needs to do what he cannot do, that’s the fixing paradigm in action.

In the pages ahead I describe how to move beyond the fixing paradigm, guided by the Nine Essentials, into a partnership with your child and the creation of a roadmap for awakening the potentials of his brain. At the heart of learning how to best help your child is this basic truth: If he could, he would; if she could, she would. If he could sit, he would sit; if she could talk, she would talk. Discerning and respecting what your child can or cannot do at any given moment is a key factor in helping them move beyond their limitations. Accepting the basic truth that if he could, he would; if she could, she would, becomes an exciting and inspiring opening when applied in light of the Nine Essentials.

At the heart of learning how to best help your child is this basic truth: If he could, he would; if she could, she would.

The Nine Essentials are tools that your child’s brain, and every brain, requires to wake up, become a powerful learner, growing and evolving on all levels. The Nine Essentials help you establish the ideal internal environment for your child’s brain to do what it must do to develop and grow, creating the unique patterns and solutions that his particular special needs require.

Everything we do—walking, talking, thinking, feeling, relating to others—is made up of the millions of random experiences that we have had since the moment of our conception. All of our activities are made possible because our brains organize our experiences as dynamic, ever-changing patterns that direct those activities, whatever they might be. All of our activities are made possible because our brains organize our experiences as dynamic, ever-changing patterns that direct those activities, whatever they might be.

All of our activities are made possible because our brains organize our experiences as dynamic, ever-changing patterns that direct those activities, whatever they might be.

The Necessity and Purpose of Randomness

When a child has a special need, that condition itself will limit his opportunities for having certain experiences: physical, emotional, and intellectual. For example, when a healthy infant is lying in the crib awake, his arms, his legs, his back, and his belly will twitch and move from time to time. These movements are unintentional; they are what I call random movements. When a baby’s arms are tight, immobile or spastic, spontaneous and infinitely rich and varied random actions cannot take place. Those random movements of the more typical baby may not seem like much at the time. But for the child’s brain, they provide a rich flow of experiences and information that are absolutely necessary for the brain to eventually develop controlled and effective movements and actions. These random experiences would ordinarily be generated through the child’s own random actions; they are in fact required for every child’s brain to form itself as fully as it might. It thus becomes our challenge to figure out ways for the child with special needs to get those random experiences that his condition otherwise prevents or limits him from getting on his own. Along the way, we need to remember that these experiences will become rich sources of information for his brain.

The good news is that we can create opportunities for children to have these random experiences. When forcing them to do the actions that they are presently unable to do⎯the fixing paradigm⎯we deny them the richness of information their brain needs to learn that which we’d like them to learn. By introducing randomness to the process we make it possible for the child’s brain to ultimately create better, more organized patterns for moving his arms or doing what he would otherwise not have the information to do. The child can only do this by starting with where his abilities are at the present moment.

Those random movements of the more typical baby may not seem like much at the time. But for the child’s brain, they provide a rich flow of experiences and information that are absolutely necessary for the brain to eventually develop controlled and effective movements and actions.

Moving Beyond the Fixing Paradigm

For a person wanting to help a child who is unable to crawl, it might seem perfectly logical to get down on the floor with him, put him up on his hands and knees, support him in that position, and then try to assist him in making crawling motions. To some extent, we might be successful, but just as often this doesn’t work, or at least not well. Why doesn’t it work? To put it simply, by focusing on the end result we want the child to achieve, we rob him of opportunities for having the myriad random experiences his brain requires to form the inner patterns to do that activity well; note here that these are experiences that the healthy child does get. We are thankful there’s an alternative, which I describe as the Nine Essentials in the pages ahead. By using the Nine Essentials we provide the child with the opportunities to get the random experiences and wealth of information his brain requires. The rich body of experience that the child then gathers, like a veritable galaxy of knowledge, provides that brain with what it needs not only to figure out and perform certain actions but to refine and enhance them, in short, to become an exquisite learner.

By using the Nine Essentials we provide the child with the opportunities to get the random experiences and wealth of information his brain requires.

What we know from the science of neuroplasticity—that is, the brain’s ability to reorganize itself by forming new neural connections—is that the brain uses every bit of information that it possesses in an infinite number of ways, surprising and unpredictable in their ingenuity. This underlying galaxy of knowledge, the billions of connections and patterns that the brain creates, will become the source of information to create skills and refine abilities from now into the future. The experience of crawling, of babbling, of listening to music, of catching a ball or recognizing the difference between cold and hot, will come into play in everything we do, be it physical, emotional, or intellectual. It can be helpful, in this respect, to consider everything we think, do, and feel as movements organized by our brains.

From Fixing to Connecting

The Nine Essentials move us quickly beyond the limitations of the fixing paradigm, helping any brain do its job better, and providing the underlying process from which all skills and learning emerge. The Essentials help get your child’s brain to work brilliantly, as well as, or even better than, other children’s brains, which the child with special needs will require to problem solve and figure out ways to make the impossible possible.

The Nine Essentials are tools for you to feel, see, notice, and create; more important, they will increase your own capacity to connect with and work with your child, whatever his special needs may be. You’ll expand your ability to experience the world from your child’s point of view, oftentimes lending him the capacities of your own brain to feel, to think, to differentiate, to move, and to listen.

The Nine Essentials are tools for you to feel, see, notice, and create; more important, they will your own capacity to connect with and work with your child, whatever his special needs may be.

The purpose of connecting with your child through the Nine Essentials isn’t just about being more compassionate. Nor is it about doing for your child what he cannot do himself. On the contrary, it is about providing your child with the greatest opportunity to develop real skills and a strong sense of self, to feel good about himself and to have the ability to continue to learn and grow, with a genuine sense of accomplishment and pride.

Children Learn What They Experience

Connecting in the ways described in the Nine Essentials builds on the knowledge that all children learn their experience; they don’t necessarily learn what we intend them to learn. When we drill a child in our efforts to teach him to do something he can’t presently do, the child learns his own experience of those efforts. This may very well include learning to fail at what he is attempting or acquiring bad habits at what he does learn to do. Added to this are the possible feelings of fear, inadequacy, being bad or wrong, and even anger or resentment and a sense of failing to meet another’s expectations. Any skills the child might learn in the process of being drilled include the child’s whole experience of that effort. Limitations the child experiences in this way may support his belief that he cannot move beyond those limitations.

Every child is a living, feeling, sensing, thinking, and actively learning participant in any and every ability he ever develops in his life. The effectiveness of any help we might offer depends on how successful we are at assisting the child’s own brain in creating its own unique solutions. Instead of trying to impose these solutions, as a car mechanic does when she replaces worn parts, we need to provide the child with the richness of experience he needs to feel, and from which he will form every ability, every movement, every action for himself and within his own brain. It is important to remember that these experiences have to be where the child is right now in terms of what he can already do; only then can the child connect with what he is doing as well as to his own self, if he is to make sense of the experience and progress beyond his present limitations.

The effectiveness of any help we might offer depends on how successful we are at assisting the child’s own brain in creating its own unique solutions.

Any time we try to take the child away from where he currently is in terms of his present abilities, both parent and child will immediately experience the loss of connection with the other. When that connection is lost, it is usually a signal to us that we have slipped into the role of fixer; little will be accomplished until we reestablish that connection. For instance, if I see that it is very difficult for the child to sit up, I need to pull back, at least for that moment, stop trying to force him into a sitting position. I go back to something that he is actually able to do. Through the Nine Essentials you will be able to better recognize what your child is experiencing and work with his present capacities whatever they may be, even as you are facilitating their next breakthrough.

Any time we try to take the child away from where he currently is in terms of his present abilities, both parent and child will immediately experience the loss of connection with the other.

In the pages ahead you will discover how to make this profoundly important shift, from fixing to connecting with your child. As elusive and challenging as this shift might seem, it will make a huge difference in your child’s life and in yours. Parents often remark that the possibilities that open up often border on the miraculous. Through the parents’ work with the Nine Essentials a child who seemed oblivious to his surroundings suddenly takes great interest in the people around him; a baby with brachial plexus injury (injury involving nerves to the shoulder, arms and hands) suddenly begins moving and using his arm; a child who has great difficulty solving math problems begins to understand the meaning of numbers and to everyone’s surprise starts loving his math class. The shift from fixing to connecting, which comes about through the skills of the Nine Essentials, will provide your child with rich new opportunities to feel and connect with himself and for his brain to function more and more effectively.

Through the Nine Essentials, you will learn to focus and awaken the underlying process of your child’s discovery and creation of self, which is at the heart of successful growth and development. You will learn to shift your focus away from what your child “should” be doing now according to age and known developmental stages. You will become an astute observer of the smallest changes that occur in your child and develop an increasing appreciation for these changes and how it is that from the smallest of changes the big solutions grow. We’ll examine how and why this shift is transformational for you and your child and look into the science that presently supports it.

You will become an astute observer of the smallest changes that occur in your child and develop an increasing appreciation for these changes and how it is that from the smallest of changes the big solutions grow.

As you practice the Nine Essentials with your child, you will find yourself stepping beyond your own fear, shock, confusion, guilt, and myriad other feelings you may have. As you already know, your child’s out-of-the-ordinary needs call on your out-of-the-ordinary potentials, beyond your own expectations and aspirations. The Nine Essentials make accessible what might otherwise seem beyond your reach, making the impossible possible for both you and your child, and making your time together more pleasurable and rewarding.
