# Supplementary material for: From fixing to connecting—developing mutual empathy guided through movement as a novel path for the discovery of better outcomes in autism
Source: Front Integr Neurosci. 2025 Apr 2;18:1489345. doi: 10.3389/fnint.2024.1489345 (PMC12031662; doi:10.3389/fnint.2024.1489345)
Supplement: Supplementary file 2 [file Supplementary_file_2.docx]

Chapter 3

Your Child’s Amazing Brain

We are in the early stages of a Brain Plasticity revolution.

—Michael Merzenich, PhD

From the very beginning of my work, thirty years ago, it was clear to me that the problems I was seeing with children who have special needs has to do with the brain. Regardless of the need or disability, whether it is autism or cerebral palsy or some other condition, the issue always comes back to the brain.

Our brains organize all that we do. They create order out of chaos and make sense of the constant flow of stimulation coming our way. But how does the brain do this, and what does this have to do with your child’s special needs? The first part of the answer is that everything we do, every action we take, every movement that expresses our lives, every thought and emotion is made possible by our brain’s capacity for organizing and bringing coherence to life’s endless flow of stimulation and sensation. When a child has special needs, this process is interfered with. It is here, in the brain’s capacity for organizing and making sense of itself and its world that we find our greatest opportunities for helping our child grow past her present limitations.

Our brains organize all that we do. They create order out of chaos and make sense of the constant flow of stimulation coming our way.

Random Movement Feeds the Brain

After a child is born, she is just beginning to discover that she is a separate being in the world, an individual with a body, feelings, desires and needs. She is flooded with sensations from all of her senses, from various processes going on inside her and from her own movements and interactions with her environment. This is where creating order out of chaos comes in, transforming her mostly random movements and sensations into actions that are purposeful, intentional, recognizable, and meaningful to her.

As the baby lays in her crib during the first weeks of life, her brain is just beginning to figure out what to do with her sensations and how to organize her movements and perceptions. Watch a newborn baby and what you’ll probably see is a lot of twitches, wiggles, and unintentional movements, which I call random movements. All this activity may seem aimless. What we don’t see is what is going on in the brain. And there’s a huge amount going on in there.

With each movement a rich flow of sensations is being sent to the brain, sensations from the baby’s arm as she move it across the fluffy blankets, the sensations of pressure on the child’s back, the complex of sensations that are sent to the brain by movements of the muscles, joints, and bones. When a little arm reaches out and is caught in Mother’s hand, followed by Mother’s affectionate squeeze and the soothing sounds of her voice, all of this is experienced by the child. Each sensation experienced has the potential to feel different, one sensation from another. The brain’s ability to perceive the distinct differences in these sensations is the source of information from which the brain performs the extraordinary process of organizing itself, the body, and making sense of the world. It is here that we find the greatest opportunity for helping the child who has special needs by facilitating her brain’s ability to perceive differences.

In the Beginning: Perception of Differences

The transformation from random to intentional and purposeful movement and action begins with our brain’s ability to perceive differences. How easy it is to take this miraculous ability for granted! It seems like such a simple thing. It is one of those abilities that goes on in the background of everything we do, without our having to think about it or to even know it exists. But without this ability we could do little or nothing in life. All action, all skill, actually our very survival, totally depends on it.

The child’s ability to notice differences in what she sees, hears, tastes, smells, and feels in her moving body is at the heart of the brain’s capacity for creating new neuroconnections and pathways. It is the source of information for the brain. It is from this ability to perceive differences that all future patterns are formed, be it learning to grasp a toy, learning to say Mama, walking, responding to a specific word or name, or expressing delight when Daddy comes home. When we truly understand the profound importance of this capacity, it opens up vast new possibilities for helping the child with special needs.

The child’s ability to notice differences in what she sees, hears, tastes, smells, and feels in her moving body is at the heart of the brain’s capacity for creating new neuroconnections and pathways.

Thing One, Thing Two: Help Is on the Way

Without exception, the brain of a child with special needs will require help with perceiving differences, at least in those areas relating to his or her present limitations. What might this look like in real life? I’d like to tell you the story about my work with a little girl I’ll call Kassi. Her story illustrates the results we can get when we understand what the brain needs for developing a particular movement, skill, or action and how we can provide opportunities for this to occur.

I saw Kassi for the first time when she was three years old. She had suffered brain damage at birth, which left her with severe cerebral palsy. The muscles of her arms, legs, and belly were extremely tight (spastic), and there was very little movement that she could do on her own. Whenever she tried to move, her whole body tightened up even more. When her parents placed her in a sitting position on my table (a wide, padded, very stable table that resembles a massage table) her back got very rounded, and she held her arms even more tightly to her body. It was hard watching the great effort Kassi was making just to keep herself from falling. It was obviously very scary for her. Her legs were straight in front of her, held tightly together.

After several months of Kassi’s regular weekly sessions with me, during which I employed the Nine Essentials (described in detail in the chapters to come) she had improved greatly. She gained more mobility and control in her arms and back. She sat up more comfortably and maintained her balance better. Sitting up was no longer scary. Even her speech improved and so did her ability to think. She no longer repeated the same three or four sentences over and over again, but formed independent thoughts and learned to communicate her desires more clearly.

But there was one thing that seemed out of my or Kassi’s reach, one thing that stayed the same no matter what we did. Kassi’s legs remained tight and held together all the time, even when she was inactive, as if held together by invisible straps. When I moved her legs very slowly and gently I was able to separate them and move them freely and independently. But the moment Kassi tried to move her legs on her own initiative or move in any other way, both her legs instantly became very spastic. I kept wondering, how could she be learning new ways of moving and gaining greater freedom everywhere in her body except for her legs.

Then one day, it suddenly dawned on me. Kassi did not know she had two legs. She had never felt them as separate because they always moved together as one. She had never perceived the difference between her right leg and her left leg. And difference that is not perceived does not exist. In her experience, and in her brain, she had one leg, not two. There was no “Thing One” and “Thing Two.” There was only Thing One. Clearly, anyone looking at Kassi could see that she had two legs, a left one and a right one. But her brain did not know this.

Difference that is not perceived does not exist.

Recently researchers Michael Merzenich and colleagues were able to induce cerebral palsy–like symptoms in the hind legs of rats. They did so by tying the two hind legs together at the time the rats were born so they always moved the two legs together as one. After a while, when the restrictions on the legs were removed, the rats continued moving them together as if they had only one leg, just like Kassi. Their brains had mapped the legs as one, not as two.

My realization that Kassi’s brain was mapping her two legs as one was an important breakthrough, profoundly influencing my work thereafter. It opened a vast world of new possibilities for helping children with special needs take advantage of their brains’ remarkable flexibility and capacity for remapping and changing itself by providing the conditions for the child to perceive differences where she had not perceived them before.

Let’s Play: Kassi Discovers Thing One and Thing Two

Once I had realized that Kassi’s brain had mapped her two legs as one, it became clear that she needed to somehow feel and recognize that she had two legs. But how could we bring this about? I had gotten her legs to move separately many times, but to no avail; all these exercises and sensations continued to be interpreted by her brain according to its one-leg map. I understood that it was Kassi, not me or her parents, who had to notice her two legs. For that to happen, I needed to somehow get her to take interest in, pay attention to, and perceive the two-ness of her legs.

Like all children, Kassi loved to be playful. I got out my washable nontoxic markers, placed Kassi in a sitting position with her back leaning against my chest, and then I gently brought her right leg up so she could see her knee. I tapped lightly on her right knee and when she looked in that direction I asked if she would like me to draw a picture on this knee. She said yes. Then I asked, “Shall I draw a cat or a dog?” (This is about the limit of my artistic skills.) After thinking about this for a moment Kassi said, “Dog.” I asked, “Would you like the dog to be brown or red?” She chose red. These were all questions requiring the brain’s perception of differences—exactly what was required for Thing One and Thing Two. I proceeded to draw an image of a red dog. I did this very slowly, naming each part of the image as it emerged: “Here’s the dog’s nose. Here’s one ear, and another ear,” and so on.

Kassi was transfixed, listening to my voice, watching my drawing and feeling the marker on her skin. When I was done, I helped her move her leg around to show the picture of the dog to her mom, who was in the room with us, then to show it to me, and then to look at it herself. I then put that leg down and slowly lifted her left leg.

With surprise and mock disappointment in my voice, I playfully said, “Oh, there is no dog or cat on this knee!” In that moment I could tell that for the first time Kassi realized there is another one of those over there. There are two legs, not just one. I asked her if she would like a dog or a cat to be drawn on this other knee. This time she selected a cat. So a cat it was. I proceeded to slowly and deliberately draw a cat on this knee.

The fact that there were different pictures on her two legs opened up a vast world of new possibilities for Kassi’s brain to begin transforming her one leg into two separate legs. Which picture did she want to show and to whom? Did she want the cat and dog to be close to each other, as when she pressed her knees together? Did she want them farther apart, as when I helped her spread her knees apart? How far apart should they be? Just a little bit? More? Far, far apart? The dog could touch her hand and the cat could touch Mommy’s hand, and on and on.

Soon she was able, for the first time in her life, to differentiate and move either the cat knee or the dog knee independently and intentionally by herself. She had two legs for the first time in her life. Those initial movements were somewhat stiff and jerky, with a limited range, but they were her movements; she was generating them herself!

What Kassi’s Brain Was Doing

Through our little game, which was fun and playful for her, Kassi’s brain was receiving, assembling, and perceiving differences, organizing a growing complement of sensations with finer and finer differentiation. With these changes spasticity of her legs gradually decreased and her ability to control movement improved throughout her body.

It’s important to see here that our work together was providing Kassi’s brain with opportunities to feel and perceive differences: Thing One and Thing Two—leg one and leg two—as two distinct parts of herself, leading to greater movement and control. Note that we did not exercise her legs. This was not about trying to get her to do what she should have been able to do had she not had cerebral palsy. I didn’t try to make her stand or walk. Rather we focused on her brain getting the information it needed to be able to recognize and organize the movements of her legs by helping Kassi perceive differences she had never perceived before. In a very real way, it was brain work, not leg work.

Over time Kassi continued to improve. The last I saw her, she had learned to pull herself up to a standing position, cruising slowly, walking sideways, step by step, holding onto furniture. Her thinking got clearer and better all the time. By the time she was five, people were seeing her as a very bright girl, which it turns out she was, though nobody had thought this when she was three years old.

The Making of a Duck

When everything is working well, each difference perceived contributes to an expanding source of information that the brain uses in an extraordinary process of organizing itself and the body, and in making sense of the world. The brain uses information it acquires through perceiving differences to create new connections between different brain cells; this capacity is called differentiation. Through differentiation your child’s brain grows and changes, creating the complex integrated patterns and mappings that will give her the ability to move and act in ways that are intentional, fluid, accurate, and effective.

In my seminars I often start our discussion about differentiation by drawing the outline of a duck on a whiteboard. Then I draw four or five large, bulky, amorphous shapes, and ask people to imagine putting these shapes together, as if they were puzzle pieces, to try to match the outline of that duck. Of course, creating an image that even resembles a duck with only those four or five bulky pieces is virtually impossible.

Next I draw lots of much smaller shapes on the whiteboard: tiny circles, squares, triangles, amorphous shapes, and dots. I point to all these shapes and ask my students to imagine that there are as many of these pieces as they might want for creating the image of the duck. This, of course, is easy for them to do. With all these tiny pieces you can create precisely the image of the duck or create any other image you might like.

This demonstration, I point out, can help us understand the process of differentiation and integration that occurs in the brain, leading to the development of more precise and controlled movement skills. With enough small shapes in the brain we can “draw” the movement we want to make. This same process applies to any thought we want to form or to understand what is said to us. Remember that every movement we make—physical, cognitive, and emotional—is organized by our brains. In essence, our brains create patterns that give order to all that we do. And it does so from the many different pieces it has at its disposal; this repertoire of information is generated by the perceived differences.

Recall the point in Kassi’s story when she first realized she had two legs: Thing One and Thing Two instead of only Thing One. We had found a variety of ways to play with the dog and cat pictures that I’d drawn on her knees, giving Kassi’s brain the opportunity for creating a collection of dots and shapes, a collection of many little pieces. For the first time in her life she perceived a difference between her two legs, which allowed her brain to begin differentiating the movements of her legs; her brain now had the information it needed to start the process of creating separate maps for moving them voluntarily. She now had millions of more pieces for mapping her two legs, perceiving not only how her two legs were two instead of one but also gaining what her brain would require for moving them more smoothly and more accurately, with greater ease and control.

It is important to note that all the dots and shapes her brain was forming would also serve in differentiating and creating patterns for many other movements she would learn in the future. As the brain gains greater and greater differentiation, it applies that greater collection of dots and shapes to virtually everything that it does.

From a Blur to Clear Focus

Okay, you may be thinking, I can see how this dots and shapes idea works for physical limitations like Kassi’s. But what about behavioral, emotional, sensory, and intellectual issues, such as when my child doesn’t respond to her name? And how can I make use of this information when my ten-year-old still doesn’t read, even though we’ve spent hours working with her and have tried everything? How can this help when my child gets upset and starts screaming whenever she is in a room with more than three or four people?

Some of these symptoms will be familiar to you if your child has been diagnosed as being on the autistic spectrum, or having pervasive developmental disorder, or has a sensory integration disorder. If you have noticed emotional and cognitive challenges with your child, you most likely will have also noticed physical and motor challenges. That is because the brain organizes the mind and body as one whole; it does not separate one from the other.

The brain organizes the mind and body as one whole; it does not separate one from the other.

As challenging as it might be to understand the connection between your child’s physical limitations and the brain, it can be even more elusive to figure out what the brain needs when the problems are in the areas of thinking, feeling, emotions, and behavioral issues. The good news is that the very same process of perception of differences and differentiation applies in the development of all skills—and this includes thinking, feeling, emotions, and social behavior.

To demonstrate how the power of the perception of differences and differentiation helps children diagnosed with issues involving cognition, emotions, and social behavior, I would like to tell you about Julian. I first met him when he was three years old. His diagnosis: autism. I vividly recall walking down the hallway next to him on our way to my office for the first time. Early on, my attention was drawn to the physical symptoms—that is, how he slouched forward as he walked, his feet dragging slightly on the floor.

From the start, Julian seemed to be comfortable with my presence, so I decided to ask him some simple questions. He responded readily, but his diction was slurred and hard to understand. He drooled excessively. And his sentence structure was incoherent. Everything he said was partially formed, with unfinished thoughts that seemed to hang in midair. Julian’s mother told me that he also had problems with fine motor coordination.

Once we were in my office, I noticed that Julian would pick up a toy and then, as if he’d forgotten he was holding it, he would just let it drop out of his hand. His losing track of the toy in his hand and letting just it fall to the floor, reminded me of how he began to form thoughts and then let them dissipate into nothingness. My impression was that Julian acted as if someone had placed a foggy lens at the gateway to his brain so that everything coming in appeared blurry, fuzzy, and vague to him. He was unable to perceive differences clearly enough to make sense of himself or the world around him.

Having this idea in mind, I put Julian lying on his belly on my table. I placed my left hand under his right shoulder and lifted it very gently, just a fraction of an inch or so at first. In fact, his shoulder would only move a small amount. As I lifted in this way I could see that his shoulder and back moved together rigidly as one unit. The same happened when I lifted his left shoulder. It was more like moving a block of wood than it was like moving the live human form with all its flexible joints, soft tissue, and resilient muscles.

As I proceeded to check out how well the movements of his legs, pelvis, and head were organized, it became very clear to me that Julian’s brain, for whatever reason, was not differentiating the various parts of his body enough for him to gain any sufficient degree of dexterity, strength, clarity, and control. Similarly, he was unable to differentiate the sounds and sights around him or to make any but the most rudimentary sense of language and ideas. His brain was obviously not very good at perceiving differences; clearly, he did not yet have enough dots and shapes to work with. I decided to start our work together by providing Julian with opportunities to notice as many differences as possible, starting with the movements of his body and, whenever appropriate, using words to describe to him portions of his experiences as they were happening.

Though Julian was three years old, the differentiation of his fingers and other parts of his hands were more like what one would expect in a one-month-old. The infant who doesn’t yet perceive his hand as having five separate fingers can make only a fist with his hands and let go. Julian still perceived his hand as Thing One. When he grabbed a toy, he was essentially making a fist and letting go; that was about the limit of his control.

Julian’s blur—his poor perception of differences and his lack of differentiation—wasn’t demonstrated only in his hands. It showed up in the way he walked (dragging his feet), in his drooling, in his slurred speech, and in his confused thinking when he tried to express himself. In fact, this lack of differentiation was global, evidenced in every part of his body as well as in his mind.

Through different movements I helped Julian perceive his head as Thing One and his shoulders and back as Thing Two. Then his shoulders as Thing One and his back as Thing Two. As if by magic, Julian was soon moving his back much better. He was able to arch his back with greater strength and twist from one side to the other with greater flexibility, precision, and ease.

I continued this session by lifting one of his arms, holding it at his forearm, and pointing it toward the ceiling. I then gently and playfully shook his arm so that his hand flipped back and forth at the wrist. After a few seconds I stopped. Julian waited, watching his hand expectantly, then said, “Again!” He had noticed that his hand stopped moving⎯he perceived the difference. I repeated the action and then stopped. After a second or two he once again asked for more and I resumed.

Julian’s attention was now palpable. He no longer seemed to be in another world as he had been when he first came in. Now he was right there with me, fully in the here and now, noticing himself and his experiences.

After a few repetitions, I began asking Julian which arm he wanted me to move, then followed his direction. Julian was rapidly becoming aware of his arms and this new movement in his wrists. By having him decide which arm to move next, I further empowered him to use his increasing capacity to perceive differences and awaken his awareness of self. We continued with more movement variations in this way for an additional twenty minutes or so, and then finished that lesson for the day.

The next day Julian’s mom reported that his drooling was greatly reduced. He had also initiated playing with a game that he had previously avoided because it had required greater hand coordination than he had until then and had been too challenging for him mentally. Now he could do it easily and well. All of this was evidence that his brain was doing a much better job of perceiving differences, differentiating, and organizing his actions.

Each day thereafter I continued to create opportunities for Julian to feel himself in new ways and to perceive new and more refined differences. By the fourth day, to my amazement, Julian looked at me and told me that his father was working in his office today. His diction was much clearer and the sentence complete. I asked Julian, “Do you ever play in your father’s office when he is working?” His initial response was somewhat garbled and I couldn’t make sense of what he said. It was obvious to me that Julian was doing some thinking but at that moment was unable to turn it into a coherent thought. So I asked the question again, phrasing it a bit differently. This time his response was clear and rather sophisticated. He proceeded to explain to me that his father had one office at home and another office not at home, and that he sometimes played in his father’s office at home but not at the office not at home.

I could barely contain my excitement. Julian’s ability to express a clear difference between his father’s two offices was indeed a significant change. It showed that Julian’s brain was becoming better at perceiving Thing One and Thing Two and thus was able to create order out of disorder. To go back to our duck drawing metaphor, Julian’s brain was differentiating more and more, developing a larger collection of tiny pieces. His brain was rapidly gaining information to successfully map his movement, his speaking, and his thinking.

It showed that Julian’s brain was becoming better at perceiving Thing One and Thing Two and thus was able to create order out of disorder.

Making the Shift

As discussed in Chapter 2, our first impulse when we try to help a child with special needs is often to focus our attention on getting her to do what she can’t presently do, be it exercising a spastic arm to try to make it move or by repeatedly trying to make a child have eye contact and respond to language. Most children will do their best to comply, and more often than not we see at least some degree of progress.

We certainly don’t want to neglect the child by doing nothing. However, over and over again in my practice, I see children who rather than learning what others were trying to teach them, have learned instead the experience of their limitations—what they can’t do, or what they can do only poorly. And these experiences become grooved in, making the child’s present limitations and the brain maps associated with those limitations more deeply entrenched. We always learn what we experience—that which is actually happening for us. This is different from learning from our experience.

We always learn what we experience—that which is actually happening for us. This is different from learning from our experience.

We are most useful to the child when we focus on helping her brain, through the process of differentiation, create the millions upon millions of Thing Ones and Thing Twos, which healthy children have in great abundance and which the brain requires for creating new, more complete, and well-organized mapping of action.

The shift, from focusing on what is right there in front of us (an arm that is not moving well enough, a child who has trouble understanding what she is told, or a child who is unable to roll over, sit, or walk) to focusing on helping the brain itself create the solutions can be elusive. It is of the utmost importance to make this shift in our own thinking—that is, to think in terms of what the brain needs in order to be able to form the patterns and skills for making those movements. It is the child’s brain that has to figure it out; we cannot do it for the child.

Children arrive at the ability to roll over or sit up, or do any of the things they learn to do in life, through an extensive process of differentiation and integration set in motion within the brain. Billions upon billions of Thing Ones and Thing Twos lead to the formation of millions upon millions of different brain connections (called synapses). Those connections come together in complex, dynamic, responsive, and continuously evolving patterns that eventually result in the child being able to sit up, stand, walk, and so on.

The child doesn’t plan, or know ahead of time, that she is going to sit up, roll over, or stand up, or say Mama for the first time. Rather, the first time it happens the child finds herself doing it. From the child’s vantage point, achieving such a milestone is a complete surprise, always unexpected. Our job is to help wake up the child’s brain and support this process of creation, formation, and discovery. You will learn to provide this support through the Nine Essentials, all of which can be easily incorporated into any activity or interaction you have with your child. It is all very doable, and you will begin witnessing changes right away.

Our job is to help wake up the child’s brain and support this process of creation, formation, and discovery.

You can bring the Nine Essentials into any exercise or therapy routines you may be doing with your child at home. You will discover that your child will learn and improve faster and with greater ease and, at the same time, become a happier child.
