# Supplementary material for: From fixing to connecting—developing mutual empathy guided through movement as a novel path for the discovery of better outcomes in autism
Source: Front Integr Neurosci. 2025 Apr 2;18:1489345. doi: 10.3389/fnint.2024.1489345 (PMC12031662; doi:10.3389/fnint.2024.1489345)
Supplement: Supplementary file 3 [file Supplementary_file_3.docx]

### The 9 Essentials: Map the Way from Fixing to Connecting

In the ABMNM methodology we have defined what we call the 9 Essentials. We propose that when we bring the 9 Essentials to an intervention with the child with ASD, a particular *connection* is created that greatly potentiates that child’s learning. We can bring the Essentials to the intervention process with children on the spectrum in a disciplined and predictable way, to generate what has been labelled *organic learning*. Organic learning is naturally present for TD children, particularly in the early years of their lives, when the most potent and greatest amount of learning takes place. Like familiar practices such as “mindfulness training,” or “being in the zone” in the world of professional athletics, the 9 Essentials provide guidelines that help us over time to get better and better at manifesting the conditions where the child with ASD can learn and change. The 9 Essentials are particularly accessible for practitioners because each Essential has its roots in experiences familiar to us all. Current neuroscience research supports our contention that the *Essentials* are drivers for neuroplasticity and positive brain change.

**Movement With Attention to the Feeling of Self**

Movement is present in all action and behavior. We contend that movement is a primary and integral part of all organic learning. However, movement by itself is not enough. For learning and positive changes to occur, it requires the *attention* of the child to the *sensations* associated with their movements, to what they *feel*. It requires of the practitioner to do the same, to be attuned to what they feel in themselves in order to be able to feel and notice what the child is doing so that they can join them. As can be seen in the video of J’s session, at the beginning he was doing automatic, forceful, partially reflexive, and repetitive arching movements. It was like he wasn’t there. When I joined him and connected with him through my sensing of his movements, not trying to control or inhibit them, or try to have him change what he was doing, he very quickly began noticing. He began *feeling* the sensations generated by his movements. I was not trying to teach J anything specific, yet through our dyadic ‘dance’, his brain spontaneously “woke up” to himself and his environment. He almost immediately began changing. He became more aware of his body and his environment and started interacting with me. Movement is always present and is a potent way to communicate with the child and help drive powerful outcomes. We believe that movement needs to be central to any intervention with ASD, but not as an ‘exercise’, or direct attempts to acquire or improve existing motor functions, i.e. fixing, but rather as a means to communicate with the child in ways that upgrade the functioning of their brain that can be done by applying the *Essentials*.

**Slow**

Fast, we can only do what we already know. That is how the brain works. Fast is made possible by grooving in of new neural networks through repetitions. Think of an infant staring with rapt attention at their very slowly moving hand and fingers. When they do that their hand and fingers are being mapped to their brain. To learn and master new skills and overcome limitations, the first thing to do is to slow way down. Slow helps the brain notice differences, sense, feel, and gets the child’s attention. Slow provides an opportunity for the child’s brain to get out of its rigidity and compulsivity. During the session with J, I slowed the process way down by taking the sock off his foot very slowly in an exaggerated way. He followed my actions closely, after which he began doing a cascade of things for the first time in his life. Slow requires that the therapist, teacher, or parent slow down themselves, thus feel and notice more what is happening in themselves and in the child, which allows for true connection.

**Subtlety**

A powerful way to enhance the ability of the child’s brain to perceive differences and thus have a less “noisy” brain and greater ability to learn, is to reduce the force, or the intensity, with which we interact with the child. This includes the intensity of the demands we place on the child. The more intense the incoming stimulus, the greater the added stimulus needs to be in order to perceive it. In a dark room we will immediately notice a flash light being turned on. In full day light we won’t notice the added stimulus coming from a flash light. There will need to be a very intense added source of light for us to notice a change. The implications of this neurophysiological phenomenon (known as the Weber Fechner law) are immense. A very common response when a child fails in achieving what is asked of them is to intensify the stimulation with an attempt to try and help them “get it”. The increased intensity can be in the form of forceful attempts, many repetitions, long session durations, many daily hours of intervention, and prompting. While working with J, I began by joining and following his movements, intentionally not adding any force on my end. After joining him, when I began introducing some variations, I used minimal force as long as he was following me. Intentionally using subtlety allowed me to continuously notice what J was doing and how he was changing and respond in the moment. When interacting with children with ASD, when we reduce the intensity, as described above, we are training ourselves to shift from fixing to connecting.

**Variations**

Variations are at the heart of learning and a necessity for learning. Variations generate differences to be perceived. We see enormous number of variations in movement from birth. TD children exhibit lots of spontaneous variability in every aspect of their developing skills. Without it their development will be greatly impeded. Variations provide the brain with information from which to differentiate and integrate new skills. In the lesson with J, I introduced variations around the movement of his pelvis, then shifted to the movement of his torso, then introduced variations within those movements including speed, size, and intensity. I also introduced variations in my tone of voice, and in what I said to him, all the while staying connected with him. We can think of variations as play, mistakes, creativity, or exploration. The child with ASD tends to be rigid with a limited repertoire of repetitive, grooved-in behaviors. When the child has difficulty doing, or understanding something, rather than ask them to do it the *right* way, you can instead introduce variations closely relate to what they are doing and help them do it in many different, “*wrong”* ways. This will free your own creativity and theirs and connect you to the child, and the child to you.

**Enthusiasm**

Enthusiasm is self‐generated; it is a form of amplification of something that is felt or experienced. The feeling of enthusiasm brings the experience to the foreground and thus makes it more likely to be noticed and learned. It is easy to get enthusiastic about a desired change such as when a child with ASD begins talking, or starts looking in people’s eyes. These are what most people would consider “big” changes. However, all “big” changes emerge from many “small” ones that integrated themselves into the “big” ones that we easily notice. Therefore “small” changes have equal, or bigger value than “big” ones. The “small” changes serve as the basis, or the bridge to the next “bigger” change. When the person working with the child is attuned to the fine changes, notices them, and amplifies them by feeling enthusiastic about them, it helps the child notice those changes in themselves. However, enthusiasm is not excitement, it’s not clapping or cheering, or telling the child that they are good. Enthusiasm is an *internal*, quiet and intentional process where one *chooses* to feel delighted about seemingly small changes in the child and understand their value and importance. The child feels the enthusiasm, *without anything being said*. We allow them the space to perceive and amplify the change, grooving it into their brain and developing agency in the process. Enthusiasm is a skill that can be developed and the more it is applied by the practitioner, the more potent the child’s learning process becomes.

**Flexible Goals**

Our society places great value on goal setting, including time-limits for achieving those goals. Many conventional therapies, and special education plans, are set up with goals that are to be achieved within a specific time frame. This approach falls right back onto the mechanical model and the “fixing” approach that we discussed earlier. To follow the dictates of preset goals, and tackle them as required, the practitioner is called upon to disconnect from the child’s own experience and reality, and also from the practitioner’s own internal experience. They are then left to base their process with the child on whether or not the goal has been achieved. When the child fails to achieve the prescribed goals, the practitioner often resorts to repetitions and prompting, both externally generated and imposed by the practitioner; such demands become the primary tools that are employed in trying to “make” the child “get it” and may miss the opportunity to open new possibilities for the child. The child’s own experience – the source of information from which the child learns - becomes secondary to the process. This approach risks overlooking and denying the child the underlying process by which all TD children learn – the organic learning process, the process by which babies learn to move, think, and function independently. Organic learning is the most concentrated and consequential learning that humans experience during the first years of life as seen by the rate of growth of connections in the brain during those years. The organic learning process does not include pre-set goals, or any goals we might set for the child. We propose that setting up the practitioner to try to achieve specific goals denies the practitioner as well as the child the connection they could have with each other. It is through this connection that the practitioner can meet the child’s needs a lot better, and by our experience, we found that it provides the child with the opportunity to attain significantly greater outcomes with greater speed. With the organic process, both the practitioner and the child benefit from experiencing joy, greater agency, and empowerment.

**The Learning Switch**

At any given moment the brain is either in a *learning* mode, when the learning ‘switch’ is on – or it’s not. You know that the ‘switch’ is on when the child spontaneously pays attention, shows interest, and initiates their own involvement; this is when unexpected, or unplanned for positive changes and freedoms - motor, cognitive, and social - emerge in the child. Stress, fear, pain, multiple repetitions, fatigue, hunger, and repeated failures can turn the learning switch off.. These conditions have to be taken care of before exposing the child to a learning process. With many children on the Autism Spectrum, their learning switch seems to be turned off. We propose that this happens partially due to their condition, coupled with difficult experiences they have had in past therapeutic or didactic situations. We found that when the practitioner is applying the *Essentials* within their own actions, and in their interaction with the child, the ASD child’s own learning switch usually turns on almost instantaneously. That’s why we call it a “switch.” In the beginning of the session with J, he was “not there.” By connecting with J through my use of the *Essentials*, (movement done with slow, variations, subtlety, and flexible goals) J very quickly started noticing, paying attention; at first it was only for a very brief time but these times became longer and longer as we went on. He began to relate to me and eventually played with my fingers, with clear intention and refinement.

It is important to note that there is one more condition that can turn the child’s leaning switch off. That’s when the child has just experienced a breakthrough, just figured out something new for them. This is the time to pause, to sit back and allow the new learning to sink in, to get integrated. If we try to “pile on” more new learning at this time, it will very often “erase” the new learning, and might even inhibit it from emerging in the future.

**Imagination and dreams**

The ability to “make up” and experience in our minds something that has yet to occur in the external reality is indeed remarkable. In ABMNM we often utilize imagination as part of the intervention. For example, I was working with a 3 years old child who had Charcot-Marie-Tooth disease. As she began having the ability to move her arms, I invited her to hold and hug imaginary toys of different sizes. She loved this game and responded very well. In general, it is believed that children with ASD lack imagination. This is partly based on observing their repetitive behaviors and concluding that their limited repertoire means that they lack imagination or creativity. As more adults on the spectrum have improved their communication skill, we have discovered that many have rich inner lives but were just limited in their ability to share it. Even if the ability to imagine is limited for the child or adult on the spectrum, the development of imagination, like all other skills, is dependent on the process of discrimination, differentiation and integration. In my empirical experience I have found that using combinations of the other Essentials that I’ve described above can be very effective in helping the person on the spectrum develop their imagination. Imagination can be a very powerful tool for the practitioner to employ as they work with the child. Dreams—those of the child as well as those of the practitioner -- can help open us to possibilities that we might otherwise miss. In the words of Joseph Campbell, “…the powers personified in a dream are those that move the world.” “Dreams are extremely important. You can’t do it unless you imagine it.” –George Lucas, American Filmmaker.

**Awareness**

When working with infants as young as a few days old, I noticed that the moment I connect with them in the ways presented in this article they very quickly, sometimes within seconds, stop doing what they were doing, which could be crying, twitching, flailing, etc. They become very still and attentive to what they are feeling. I suggest that they notice and feel sensations that are different from what they are accustomed to. They become aware of this without words. This way of connecting with the child, including the child or adult with ASD, through the Essentials, provides their brain with the conditions that helps it make sense of the incoming stimulation; they begin to form connections and patterns, putting in the disorder. Think of Y, how he came in highly agitated and arching involuntarily over and over again. A few minutes into the lesson he became aware of his own foot, then of me. His awareness grew to the point that he tried feeding me his apple and eventually played with my hands and fingers. I see awareness as an emergent quality that arises from movement combined with sensing and experiencing outcomes. Awareness does not require language, even though children, as they learn to speak, often times “narrate” what they are doing, clear evidence of awareness. My understanding is that their brain maps the parts of the body involved in the movement and the different relationships between those moving parts of their body. The outcomes associated with these movements also get mapped to the brain. They begin forming a universe of cause and effect, i.e., cognition. We propose that awareness of self is integral to an effective learning process. It also helps accelerate the grooving in of new learnings, greatly reducing the need for repetitions. The practitioner joins with their awareness the awareness of the child, thus creating a loop, a dance, if you will, resulting in a vibrant process of learning and change for the child. And often, for the practitioner, too!

For more information regarding the thinking behind the 9 Essentials and the science validating them please see

Baniel, A. (2012). Kids Beyond Limits: The Anat Baniel Method for Awakening the

Brain and Transforming the Life of Your Child with Special Needs. New York, NY:

Penguin.
