# Supplementary material for: From fixing to connecting—developing mutual empathy guided through movement as a novel path for the discovery of better outcomes in autism
Source: Front Integr Neurosci. 2025 Apr 2;18:1489345. doi: 10.3389/fnint.2024.1489345 (PMC12031662; doi:10.3389/fnint.2024.1489345)
Supplement: Supplementary file 4 [file Supplementary_file_4.docx]

**Report from the Director of Education where the ABClassrooms (Anat Baniel Classrooms) program was implemented**

I wanted to clarify the discussions we are having about the impact of Anat Baniel Classrooms.

The key measure for me is Serious Behavior Incident Data.  This data is charted individually by Principals year by year.  Serious Incidents are for the entire school.  They are issues that go to a Principal for resolution.  Issues can be violence or student outbursts that prevent classes from continuing and violence against staff.  These incidents have in common that they may result in a child being removed from School for a period of time.

Father Gorman School where we began our Anat Baniel Classrooms in 2019-2020

| Academic Year | Total | Comments |
| --- | --- | --- |
| 2018-2019 | 80 | A large portion were based around key students who have left.  Two VTRA’s that were highly unusual and very lengthy.  Both children out for months. |
| 2019-2020 | 60 | I anticipate this would have been higher as we shifted to online learning. |
| 2020-2021 | 48 | Notable decrease |

At Father Gorman it is notable that in two years of practice we see a decrease of 40% in serious behavioural incidents.

At Mother Teresa School the impact is even more clear

| Academic Year | Serious incidents | Behaviour Incidents | VITRA (incidents requiring social services and or Police Intervention |
| --- | --- | --- | --- |
| 2018-2019 | 14 | 11 | 1 |
| 2019-2020 | 10 | 4 | 4 |
| 2020-2021 | 5 | 4 | 0 |

The issue here is that VITRA incidents are those that require the involvement of either the police or Social Services.  You will note that these incidents have been eliminated in 2020-2021 and that incidents of a serious nature are 1/3 of the normal numbers in the baseline year, 2018-2019.

I draw 2 conclusions from the schools where the Anat Baniel Classroom program has been active:

If we alter the experience of a challenged population, we alter the learning environment as a whole.  The learning environment is more calm and the challenged population can access learning rather than focus on the barriers to their learning.  This decreases violence in schools and the issues that result in a student being referred to the principal for disciplinary issues.  Key is that high needs children are less elevated where we see Anat Baniel Classroom in action.

As Educational Assistants learn to move from fixing to connecting their expertise serves the school as a whole.  Professional Development for the Educational Assistants was the primary goal of this program.  It is clear that in growing their competency we decrease student time away from school and the learning readiness of the classroom environment.

There is data to support this provincially as well.  In a year when COVID impacted learning, LCSD did not see the learning loss that is so prevalent, locally and across the United States and Canada.  I offer as evidence of this claim the Grade 3 reading test results.  Reading tests at this level has the highest positive correlation to long term graduation statistics.  It is also related to other factors, incarceration, earnings potential and others.  In this case, our *At Grade Level Reading Results* in 2020-2021 increased 4% to 95%.  Our baseline year was less than 90%.  Again, there is evidence that the Anat Baniel Classroom has had a positive impact system wide.

Finally, there is another school wide statistic to consider.  Student injuries

Within student injuries reduced from 442 in 2019 to only 152 in 2020. Staff injuries reported reduced to 8 from 10 in 2020. School was closed for 33% of the days but student injuries were reduced by 66%.  This is half as many injuries as the prior year.

So, in a school system where the Anat Baniel Classroom was instituted we can say with confidence that there were

- Half as many student injuries
- An increase in reading results system wide
- A decrease of at least 40% but up to 66% of serious incidents referred to the Office for resolution in ABC schools
- A near elimination of Social Services and Police involved incidents in one ABC school

These are significant impacts.

It is important that we spend time to study this evidence further.  A system wide impact of undertaking the Anat Baniel Classroom is clear anecdotally, but further work supported by granting agencies would enable us to clear attribution errors and tie impacts of Professional Development of Educational Assistants as well as the direct interventions for high needs students to the success of the entire school.

We continue to appreciate your partnership Anat, it is humbling to be a part of the beginnings of something that may change the world of education.

Nigel McCarthy

Director of Education

Lloydminster Catholic School Division
