# Supplementary material for: From fixing to connecting—developing mutual empathy guided through movement as a novel path for the discovery of better outcomes in autism
Source: Front Integr Neurosci. 2025 Apr 2;18:1489345. doi: 10.3389/fnint.2024.1489345 (PMC12031662; doi:10.3389/fnint.2024.1489345)
Supplement: Supplementary file 5 [file Supplementary_file_5.docx]

**RESEARCHING A GROUND-BREAKING, NOVEL APPROACH TO LEARNING AND EDUCATION IN SCHOOLS AND IN CHILDREN WITH ASD:**

**Background, Preliminary Outcomes, Technical Summary**

**Introduction/Abstract:**

6 children with significant special needs, from the Lloydminster Catholic School District (LCSD) in Alberta Canada received a total of 20 one-on-one sessions each from an Anat Baniel Method®NeuroMovement® ABMNM®) practitioner over a period of 6 months. Progress was documented through video capture and written anecdotal observation and self-reporting of the parents. 2 of these children were fitted with wearable sensors that provide information on the brain’s quality of organization and change in response to the intervention. The practitioners were also fitted with the same wearable sensors and the same data was collected from them as they worked with the child. The data is awaiting analysis at the Torres Sensory Motor Integration Laboratory at Rutgers University. The analysis will be of the individual changes in brain activity as they correlate to gained learning in the child, as well as analysis of the correlation between changes and quality of brain activity of the practitioner’s brain to changes and quality of brain activity in the brain of the child as they change. This research was conducted as part of the Anat Baniel Classroom (ABC) Program outlined below which has already produced remarkable learning and behavioral outcomes in the 2 schools in which it was implemented.

**Background**

Schools, and especially public schools, are facing growing challenges due to an ongoing surge in the number of children diagnosed with autism, ADHD, undiagnosed developmental delays and behavioral challenges. Significant resources are put into trying to help these children integrate into the classroom with minor results, and often failure. Due to the rapidly growing number of such children in the schools, the disruptions can become unmanageable for the teachers, the aides, and interfere with the rest of the children’s learning.

In 2019 Anat Baniel was approached by the school board of Lloydminster Catholic School Division* (LCSD) in Alberta Canada and the Director of Education, (Superintendent), Nigel McCarthy, with a request to bring the Anat Baniel Method®NeuroMovement®(ABMNM®) into their 6 schools. His goal was primarily to provide the aides, called in Canada "Educational Assistants"(EAs), with potent tools to help the challenged children become effective learners, have them better integrate with the rest of the class, and be less disruptive to themselves and others. Prior to approaching Anat, he had observed that despite the EAs dedication and hard work, they were ineffective in helping the children learn and participate in the classroom. Their role was mostly to try to control and contain the challenged child, often unsuccessfully. The Director also realized that the EA’s had the least amount of training compared to all other professionals in the school while being tasked with one of the most challenging roles.

**Lloydminster Catholic School Division (LCSD) in Alberta, Canada**

LCSD is a publicly funded school division offering an inclusive learning environment. The demographic of their student population includes Canadian nationals, First Nation children, children for whom English is a second language, children with Autism and ADHD, and children with other physical and cognitive challenges.

**The Anat Baniel Classroom Program (ABC program):**

We ran our program for 300 children from kindergarten to grade 6 through 2019-2022 school years in two of the 6 schools. We aimed to help bring about a division-wide transformation so that the principles and applications of the Anat Baniel Method®NeuroMovement® will be understood and adopted by the teachers, the school principals, the administration and the leadership, thus creating a consistent approach and environment that supports the EA's in their very challenging task.

We introduced the NeuroMovement process school wide, starting with the whole teaching staff, administrative staff, school principals, EA's, and therapists. We taught a short NeuroMovement lesson to the children and their teachers in each of the classrooms and had the teachers continue teaching the NeuroMovement lesson classes from pre produced animated videos.

**Parallel NeuroMovement Research Program**

6 children with significant special needs, from the LCSD schools participating in the ABC program received 20 private one on one sessions each from an Anat Baniel Method®NeuroMovement practitioner over a period of 6 months. Progress was documented through video capture and written anecdotal observation. In addition, owing to progress in wearable sensors that monitor physiology and neurological and muscular activity in real time, 3 of these children, as well as the practitioner that was working with them, were fitted with wearable sensors. Data has been collected that is waiting to be documented and analyzed.

*Why NeuroMovement?*

NeuroMovement offers tools that are harmonious with brain functioning. Using these tools to connect with children where they are currently at, and providing conditions that facilitate learning, contrasts with a more prevalent approach to education which aims to make the child do what they cannot yet do or memorize facts and details that they do not understand because these skills or ideas are “age appropriate”. As Anat Baniel says, “If they could, they would”. Anecdotal evidence supports the view that NeuroMovement upgrades the functioning of the child’s brain to be a more potent learning brain that is better at figuring things out.

*Why Dr Elizabeth Torres?*

Due to the innovative work of Dr Elizabeth Torres of Rutgers University, this project provided a unique opportunity to measure changes in the child’s brain and its correlation to the implementation of ABMNM and to the observable changes in the child.

The research method, developed by Professor Torres tracks the USPO patented [1-3] *Micro-Movement Spikes* (MMS) which are the subtle and constantly fluctuating motor patterns that generate sensory signals that are not consciously perceived, at micro- to sub-second timescales. These *Micro-Movement Spikes* can be measured through the wearable sensors and be analyzed through Dr Torres's algorithms, adding substantial amount of information about motor, perceptual and cognitive functioning and maturation.

Professor Torres’s innovations offer a general unifying approach to movement and behavioral analyses. *For the first time*, her methods provide the means to capture data through sensors, that can measure the subtlety of the ABMNM® approach and document its effectiveness and its impact on the brain's quality of functioning. As the NeuroMovement intervention takes place, we posit that the brain constantly and minutely recalculates its motions and reorganizes itself, thus creating new connections. Data from the sensors collected during the sessions, together with video capture of the sessions, and accompanying written documentation are awaiting analysis in Rutgers. We postulate that this data already collected will precisely quantify these changes through combining the sensors from the Torres Sensory Motor Integration Laboratory with Anat Baniel Method®NeuroMovement®.

**Preliminary Administrative Data from the ABC Program During the 2019-20 and 2020-21 School Years**

The ABC program began in 2019 and continues to run in LCSD. Thus it includes the period when learning outcomes were severely negatively impacted by the Covid-19 pandemic both in the US and Canada. However, in contrast to the national trend, as seen below, the schools enrolled in our program had significant gains in learning outcomes and reduction in behavioral problems

There is already powerful anecdotal evidence of the effectiveness of the ABC program. In the words of the Director of Education:

“As Educational Assistants learn to move From Fixing to Connecting and employ the 9 Essentials of ABMNM® their expertise serves the school as a whole. Professional Development for the Educational Assistants was the primary goal of this program. It is clear that in growing their competency we decrease student time away from school and the learning readiness of the classroom environment has substantially increased. There is data to support this provincially as well. In a year when COVID impacted learning, LCSD did not see the learning loss that was so prevalent locally and across the United States and Canada (as evidenced by National Assessment of Educational Progress <https://www.nytimes.com/2022/10/24/us/math-reading-scores-pandemic.html>) I offer as evidence of this claim the Grade 3 reading test results. Reading tests at this level have the highest positive correlation to long term graduation statistics. It is also related to other factors such as incarceration and earnings potential. In this case, our Grade Level Reading Results in 2020-2021 increased by 4% to 95%. Our baseline year was less than 90%. Again, there is evidence that the Anat Baniel Classroom program has had a positive impact system wide.” In addition there were hugely significant decreases in serious behavioral incidents and a 50% decrease in student injuries in both schools combined.

**TECHNICAL SUMMARY**

The purpose of this study is to digitally characterize the Anat Baniel Method®NeuroMovement® (ABM®NM®) during sessions involving children with learning challenges. To that end, we will use a combination of commercially available wearable biosensors and research grade sensors, to register biorhythmic data from the dyad composed of Anat Baniel and the child, as therapeutic sessions evolve. (We also measure the movement/brain activity of the therapist as they work with the child which can demonstrate the relationship between the two and be useful in training therapists.) The data will then be analyzed by the Rutgers University Professor Elizabeth Torres, PhD, using her patented analytical methods and novel experimental assays, which have been validated in thousands of participants across the human lifespan. This work has been published in over 100 peer-reviewed scientific publications, and scholarly books on neurodevelopmental, neurodegenerative, and rare disorders of genetic origins as well as in professional sports and the performing arts.

1. A ***micromovement spikes (MMS)*** approach to both movement therapy and mathematical movement analysis/modelling can be wedded to show levels and trajectories of change in ontogenetically defined movements classes ranging from spontaneous to intended, at subtle but critical levels of efficacy *hitherto neither perceived nor quantified*.

| 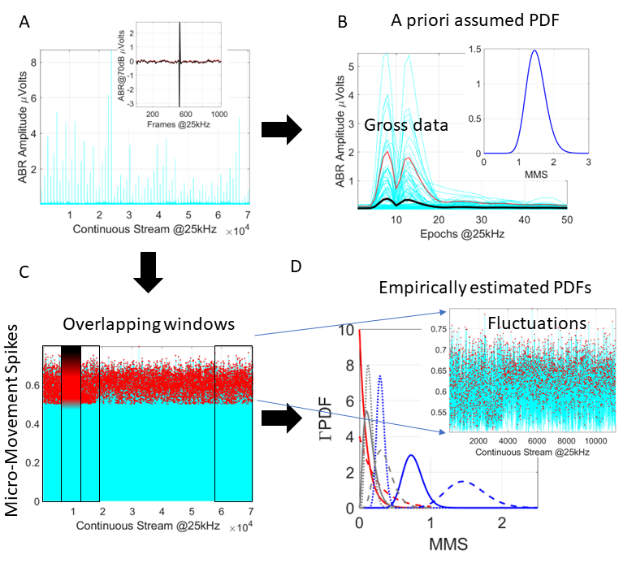 |
| --- |
| **Figure 1**. The patented Micro-Movement Spikes (MMS). (A) Sample biorhythmic activity registering responses to sound from the neonatal brainstem at 25KHz. (B) Traditional approach to data analyses across scientific disciplines assume normally distributed data and average across epochs under such normality assumption. They also implicitly assume stationarity and linearity of the process under a priori imposed parametric models. Mean activity (black curve) and two-standard deviation envelope enclose the data currently considered for analyses, while wasting as gross data or superfluous noise, the rest of the fluctuations. (C) The US- and EU-patented MMS approach considers instead fluctuations away from an empirically estimated mean under no such assumptions of normality, linearity, and stationarity in human biorhythmic data. Continuous analyses of data that is windowed according to sampling resolution and tailored to the phenomena under consideration is then performed to reveal (D) A family of distributions underlying such otherwise wasted fluctuations. This family is unique to each person and reflects the autonomous and/or guided rates of learning and adaption of the person’s nervous systems in response to development, growth, disease and treatments. |

1. The integration of Baniel’s with Torres’ methods will provide a multilayered approach to motion capture and analyses [12], *not only* to capture individual progression and progression of the Baniel-patient dyad, but also to address progression of a group session (including potential increase of synchronization therein), should the opportunity arise for group therapy.

| 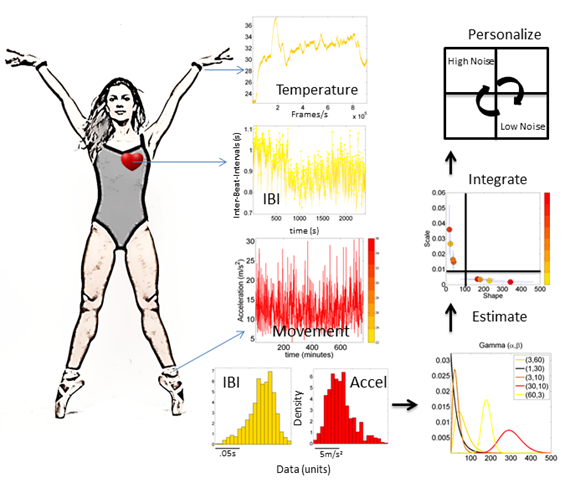 |
| --- |
| **Figure 2**. Multilayered approach to MMS analyses using non-invasive wearable biosensing technologies that are commercially available today. From head to toe, we can simultaneously track a multitude of biorhythmic time series to determine the stochastic trajectories of treatments’ outcomes and nervous systems function. US- and EU-patented technology from the Torres Sensory Motor Integration Lab (T-SMIL) can then be used to determine physiologically relevant activities and distinguish autonomic from spontaneous, from volitionally controlled levels. |

1. Since the methods are patented, licensing-ready, as well as applicable across all biorhythmic and time series data and can be embedded in commercially available technologies, the proposed approach is broadly scalable and actionable.

Upon our proof-of-concept characterization of ABMNM we can then determine latencies for feasible near-real time biofeedback to the therapist and/or child or adult and develop Apps that inform on demand, to both the clinician and patient, about the states of biorhythmic activities, adaptive levels, and physiological motor learning states.

**CROSS-DISCIPLINARY TEAM FOR A PROOF-OF-CONCEPT DEMONSTRATION**

***Anat Baniel, MS*** *– Four-plus decades of experience working across spectrums ranging from many types of often severe inborn and acquired neurological disorders and injuries to upping the games of world-class athletes and musicians.*

***Martha Herbert, PhD, MD*** *– A board-certified Neurologist with over 40 years of academic and basic research including over 20 years on the faculty of the Neurology Department at the Massachusetts General Hospital, Harvard Medical School and its Martinos Center for Biomedical Imaging.*

***Elizabeth Torres, PhD*** *– Computational cognitive neuroscientist in the areas of sensory-motor integration, perception, and cognition, who over two decades has developed and validated a range of standardized assays to objectively evaluate daily and longitudinal treatment’s effectiveness across vital functionality, affording the precision of personalized analyses, inference, and interpretation of results. See* [*https://sensorymotorintegrationlab.com/publications*](https://sensorymotorintegrationlab.com/publications)*.*

**Referenced link:**

1. Torres, E.B., *System and Method for measuring physiologically relevant motion*, in *Google Patents*, USPTO, Editor. 2017, Rutgers The State University of New Jersey: US. p. 1-21.

2. Torres, E.B., *System and method for determining amount of volition in a subject*, in *Google Patents*, USPTO, Editor. 2017, Oct. 19, Rutgers The State University of New Jersey: US. p. 15.

3. Torres, E.B., *Methods for the diagnosis and treatment of neurological disorders*, in *Google Patents*, USPTO, Editor. 2018, Rutgers State University of New Jersey: US. p. 38.

11. Bermperidis, T., et al., *Optimal time lags from causal prediction model help stratify and forecast nervous system pathology.* Sci Rep, 2021. **11**(1): p. 20904.

12. Torres, E.B., *Objective biometric methods for the diagnosis and treatment of nervous system disorders*. 2018, London: Academic Press. x, 568 pages.
