# Supplementary material for: From fixing to connecting—developing mutual empathy guided through movement as a novel path for the discovery of better outcomes in autism
Source: Front Integr Neurosci. 2025 Apr 2;18:1489345. doi: 10.3389/fnint.2024.1489345 (PMC12031662; doi:10.3389/fnint.2024.1489345)
Supplement: Supplementary file 6 [file Supplementary_file_6.pdf]

VIDEOS 2-5 – FM (Feldenkrais Method)

FM Video 2: Gabriel walking before the sitting ATM

https://vimeo.com/1004959348/2f5f31f818

FM Video 3: Gabriel in sitting jumping on the butt ATM

https://vimeo.com/1004959365/0eab7d0932

FM Video 4: Gabriel walking after the sitting jumping ATM

https://vimeo.com/1004959394/4605746479

FM Video 5: Lesson with Yochai

https://vimeo.com/1004959418/a9dd68c1ff
