# Supplementary material for: From fixing to connecting—developing mutual empathy guided through movement as a novel path for the discovery of better outcomes in autism
Source: Front Integr Neurosci. 2025 Apr 2;18:1489345. doi: 10.3389/fnint.2024.1489345 (PMC12031662; doi:10.3389/fnint.2024.1489345)
Supplement: Supplementary file 7 [file Table_1.docx]

**Supplementary Material**

| Title | From fixing to connecting—developing mutual empathy guided through movement as a novel path for the discovery of better outcomes in autism. |
| --- | --- |
| Authors | Anat Baniel1†, Eilat Almagor2†, Neil Sharp3*‡, Ohad Kolumbus2‡ and Martha R. Herbert4,5‡§ |
| Affiliations | 1. Anat Baniel Method, Inc., San Rafael, CA, United States, 2. The Jerusalem Academy of Music and Dance, Jerusalem, Israel, 3. ABM® NeuroMovement® Center of Marin, San Rafael, CA, United States, 4. Higher Synthesis Foundation, Lobelville, TN, United States, 5. Complementary Healthcare Advanced Research and Leadership Institute, Life University, Marietta, GA, United States |
| Category | Hypothesis and Theory |

**TABLE OF CONTENTS:**

**LIST OF SUPPLEMENTARY MATERIALS**

**In order of appearance in article**

| **Section in Article** | **Type** | **Label** | **File Name** |
| --- | --- | --- | --- |
| 1.1 Movement | Table | Supplementary Table 1 | Table of Contents: List of Supplementary Materials |
| 1.1 Movement | ABMNM-Document | Supplementary File 1 | From Fixing to Connecting (Kids Beyond Limits: Chapter 2) |
| 2.1 Part one: case study, Jonathan | ABMNM-  Video 1 | Supplementary Video 1 | ABMNM Case Study Video  <https://vimeo.com/1004959090/1453c9d9d5>   1. Lesson 1 – 6-minute excerpt from a 22-minute session 2. Excerpt from testimonial from mother after seven 30–45-minute sessions in 2 weeks 3. 1 minute clip of lesson with Anat during a practitioner training course 6 weeks after initial consultation 4. Follow up with J aged 9 years |
| 2.2.3 The “noisy” brain | ABMNM-Document | Supplementary File 2 | Your Child’s Amazing Brain (Kids Beyond Limits: Chapter 3) |
| 2.3 Part three: the nine essentials | ABMNM-Document | Supplementary File 3 | “The Nine Essentials” more fully described |
| 3.3.1 A Lesson with Gabriel | FM: Video 2 | Supplementary Video 2 | FM Video 2: Gabriel walking before the sitting ATM  <https://vimeo.com/1004959348/2f5f31f818> |
| 3.3.1 A Lesson with Gabriel | FM: Video 3 | Supplementary Video 3 | FM Video 3: Gabriel in sitting jumping on the butt ATM  <https://vimeo.com/1004959365/0eab7d0932> |
| 3.3.1 A Lesson with Gabriel | FM: Video 4 | Supplementary Video 4 | FM Video 4: Gabriel walking after the sitting jumping ATM  <https://vimeo.com/1004959394/4605746479> |
| 3.3.1 A Lesson with Yochai | FM: Video 5 | Supplementary Video 5 | FM Video 5: Lesson with Yochai  <https://vimeo.com/1004959418/a9dd68c1ff> |
| 4.1 ABMNM research | ABMNM Research- Document | Supplementary File 4 | “Report from the Director of Education where the ABClassrooms (Anat Baniel Classrooms) program was implemented” |
| 4.1 ABMNM research | ABMNM Research- Document | Supplementary File 5 | “RESEARCHING A GROUND-BREAKING, NOVEL APPROACH TO LEARNING AND EDUCATION IN SCHOOLS AND IN CHILDREN WITH ASD:  Background, Preliminary Outcomes, Technical Summary” |
